# Uncovering how transport access reduces deprivation: When colocation misleads

**DOI:** 10.1073/pnas.2532730123

**Published:** 2026-04-28

**Authors:** Surabhi Ojha, Anupriya Anupriya, Daniel Hörcher, Daniel J. Graham

**Affiliations:** ^a^Department of Civil and Environmental Engineering, Imperial College London, South Kensington, London SW7 2AZ, United Kingdom; ^b^Corvinus Institute for Advanced Studies, Corvinus University of Budapest, Budapest 1093, Hungary

**Keywords:** urban economics, transport networks, causal inference

## Abstract

Since transport access determines who can reach jobs, education, healthcare, and community life, governments increasingly use accessibility improvements to reduce deprivation and tackle social exclusion. Yet whether better access causally reduces disadvantage remains uncertain because observational analyses struggle to separate cause from context, and because accessibility itself can be measured in many, nonequivalent ways. Two challenges follow: i) widely used measures of accessibility, cumulative-opportunity, gravity, and random-utility may yield conflicting maps of accessibility and; ii) estimates from observational data are vulnerable to confounding. This paper conducts a London-wide assessment that a) compares widely used accessibility measures, and b) applies instrumental-variables (IV) estimation with road-safety-based instruments to address confounding and identify the causal effect of accessibility on deprivation. Using neighborhood-scale accessibility and the 2019 Index of Multiple Deprivation (IMD, proxies deprivation, and more broadly, social exclusion), we report two main findings. First, although accessibility rankings are broadly consistent across measures, gravity and cumulative opportunity measures display similar linear behavior, in contrast to the strong nonlinearity of the random-utility measure. The choice of measure affects not only how accessibility is represented, but also the variation retained for empirical analysis. Second, simple correlations suggest that accessibility and deprivation colocate, whereas causal estimates indicate a consistent, beneficial effect: Improvement in accessibility leads to lower deprivation, with magnitudes differing across IMD domains. From a policy perspective, this highlights the importance of grounding transport investment decisions in causal evidence and considering a range of measures to understand how accessibility improvements may help reduce disadvantage.

## Significance

There is growing interest in how cities can use transport to reduce disadvantage. A central challenge is measurement: Widely used “accessibility” measures often point to different neighborhoods as underserved, leading to conflicting policy signals. Using a citywide dataset for London, we i) compare common access measures side by side to show how metric choice reshapes the map of accessibility, and ii) contrast simple statistical associations with a causal statistical design that separates access from other neighborhood differences. We find that better access is linked to lower deprivation, yet the choice of measure substantially alters which areas are identified as priorities for intervention. These results can help cities target investments where improved access is most likely to reduce disadvantage.

Transport networks are central to the functioning of cities (1), providing the physical means by which people connect to jobs, services, and social opportunities (2), while simultaneously shaping urban structure and influencing where people live (3–7), work (8), and interact (9). Although transport networks can enable mobility and foster opportunity (10, 11), their uneven distribution within wider land use systems is frequently recognized to have reinforced segregation and inequality (12, 13), constraining daily mobility (14), limiting access to employment and essential services, and exacerbating disadvantage in already marginalized communities (15–17). Physical infrastructure can also become a barrier: Major highways, arterial roads, and rail corridors induce suburbanization, fragment neighborhoods, reduce opportunities for social interaction, and perpetuate segregation (18–22). At the same time, transport investments are often promoted as drivers of economic development and poverty reduction, with their potential to advance agendas of sustainable and inclusive urban growth (23) and to foster interaction across diverse social groups (1, 24). Yet, evidence on their equity impacts remains contested: While some studies find that new links improve accessibility and stimulate local opportunities (25, 26), others highlight unintended consequences such as rising land values (27), gentrification (28–31), displacement, and the reinforcement of sociospatial inequalities (32). This paper contributes to this debate by providing causal, policy-relevant evidence on the equity impacts of access to transport.

Transport equity refers to a distributive justice framework that examines how social, economic, and governmental institutions influence the allocation of transportation-related benefits and burdens across society (33). Its evaluation is underpinned by a set of normative principles (34). Horizontal equity demands equal provision across groups, while vertical equity emphasizes allocation according to need (35).

Sufficientarian approaches prioritize lifting those below a minimum threshold of access, whereas egalitarian perspectives aim to equalize opportunities across all groups (36, 37). These principles are commonly operationalized using distributional statistics, such as Lorenz curves (see *SI Appendix* section E and Fig. SI8 for a demonstration of Lorenz Curves in this paper), Gini coefficients, and Palma ratios(17, 38–41), or by mapping transport accessibility against population characteristics to reveal spatial inequalities, including more detailed, survey-informed approaches that translate individual-level mobility constraints into spatially explicit measures of unmet transport needs (42). While these methods are useful in assessing the distribution of transport services and assessing gaps in service provision, they provide limited insight into the mechanisms through which it may reinforce disadvantage.

The social consequences of transport systems are best understood through the lenses of social exclusion and deprivation. Social exclusion describes the institutional and spatial processes that limit participation in economic, social, and civic life (16, 43), while deprivation represents their material outcomes across domains such as income, health, and housing (44). Despite these conceptual connections, there is limited consensus on how accessibility shapes deprivation or social disadvantage, with theory and evidence pointing to multiple, sometimes opposing mechanisms.

**Fig. 1.**
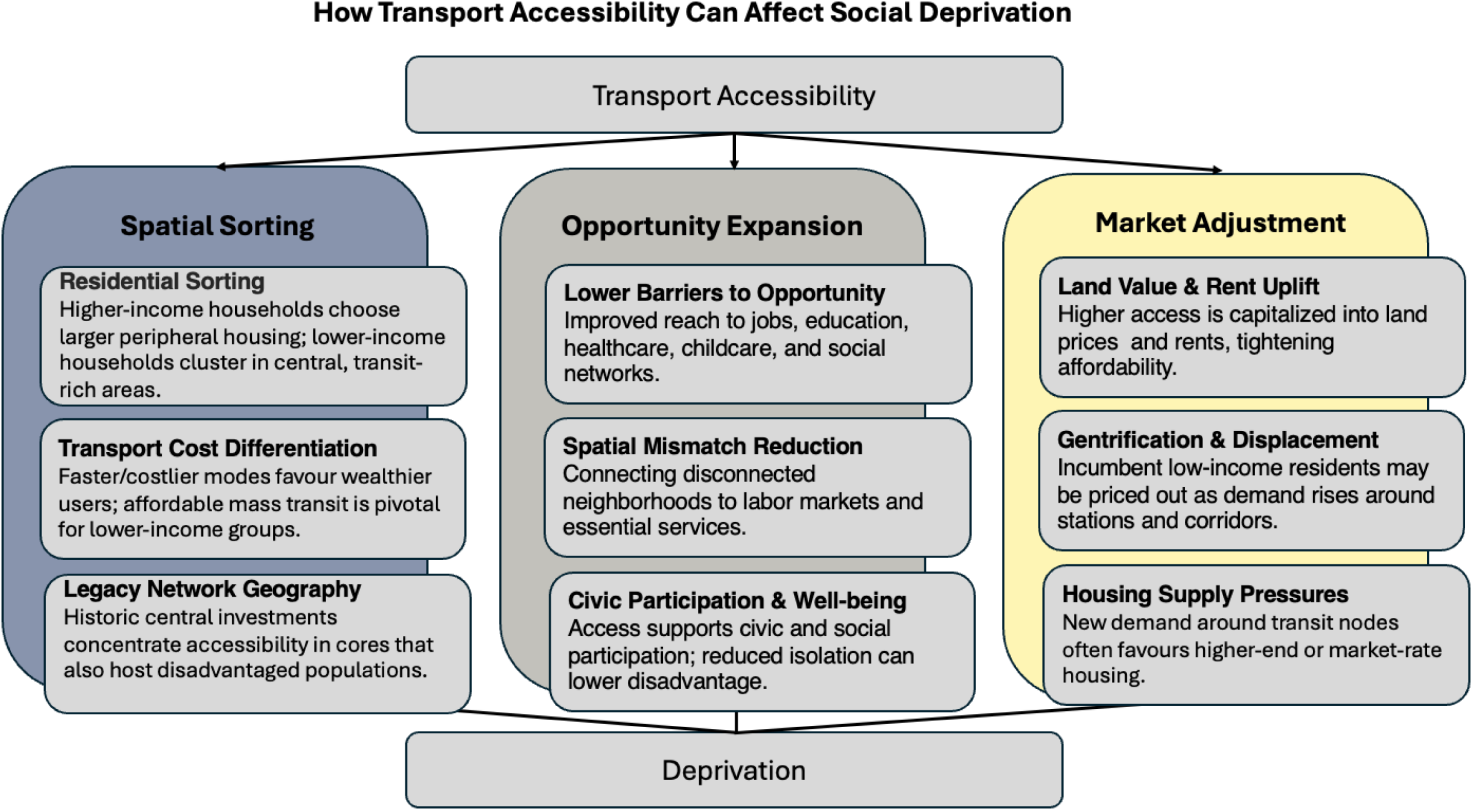
How transport accessibility can affect deprivation. The framework distinguishes three families of processes: spatial sorting, which shapes residential location patterns; opportunity expansion, which reduces barriers to work, services, and participation; and market adjustment, which reflects the response of land and housing markets. Together, these processes illustrate how accessibility can reinforce, reduce, or relocate deprivation depending on context.

The contested links between access and deprivation can be organized into three channels (Fig. 1). First, spatial sorting processes describe how income differences, legacy transit investments, and travel costs shape residential location. Since lower-income residents are less likely to own cars, proximity to high-capacity transit can draw them to central, accessible locations, sometimes alongside crowding. Where central amenities are weaker, higher-income households may prefer peripheral housing, leaving poorer households in transit-served cores (45); where amenities are strong, richer households may outbid others for central locations (7). In short, sorting can make deprivation coincide with high access or push it outward. Second, opportunity expansion processes emphasized in the urban planning literature view accessibility as a lever to reduce disadvantage: better connections lower barriers to employment, education, healthcare, and participation, mitigating spatial mismatch between deprived neighborhoods and centers of activity (2, 8, 46). These theories emphasize the expansion of available opportunities of disadvantaged groups and can reduce the intensity of deprivation, even if its spatial distribution remains uneven. Third, market adjustment processes add feedback loops to the access-deprivation link. Urban economic models emphasize the housing–commuting trade-off (4–6): Transport-rich areas offer savings for households reliant on public transport but command higher housing costs (3, 7). Thus, accessibility gains are often capitalized into land values, raising rents and prices (27), which can trigger gentrification and displacement, relocating lower-income households to more peripheral, lower-access areas (47). Taken together, these channels imply that accessibility has no single relationship with deprivation: It can reinforce existing patterns, reduce disadvantage by expanding opportunity, or displace deprivation to new locations depending on context.

Empirical evidence on the transport- deprivation link, and in turn, implications for transport equity remain fragmented. Across cities and studies, conclusions often depend on how deprivation or some indicator of disadvantage is framed and operationalized. In Beijing, higher-education groups enjoyed more equitable access to high-skill jobs than lower-education groups did to low-skill jobs (48). In Montréal, deprived neighborhoods paradoxically exhibited the greatest transit job access (49). In the United States, large city transit systems frequently provide higher job accessibility for both low and high income groups while excluding middle income households (41). Across Europe, findings also vary depending on whether accessibility is measured by the total number of reachable opportunities or by their diversity (40). Together, these examples illustrate that different definitions and metrics of disadvantage can yield divergent, even contradictory, conclusions, revealing that transport equity is not a fixed outcome but a function of how it is conceptualized and measured.

Empirical evidence is further complicated by methodological design. Results often hinge on whether studies compute mere correlations or identify causal effects. Associational analyses that relate accessibility levels to deprivation reveal stark spatial inequalities (16) yet risk conflating transport with broader socioeconomic processes (50–52). By contrast, causal designs, for instance, quasi-experimental evaluations (53–55), aim to isolate transport’s independent contribution and can reach different conclusions.

Against this backdrop, disagreements in findings also reflect how accessibility is measured. Early work from geographers and planners tracked access to transport using variables that measure the availability and quality of transport services, such as density of service kilometers, speed, capacity, frequency, and coverage (56). At the same time, a parallel strand defined opportunity-based access, that is, the ease of reaching jobs, services, and amenities (2, 46). These ideas also shaped critiques of economic cost–benefit analysis in transport, challenging its focus on time savings (57) as a primary source of benefit. The urban planning literature reframed benefits in terms of the opportunities transport networks make reachable (58).This shift has been accompanied by growing calls to embed accessibility measures directly into transport investment decision-making, particularly in long-range planning and project appraisal (59).

Three families of accessibility measures now dominate practice. i) Cumulative (isochrone) counts tally opportunities within a travel-time threshold, which is intuitive and policy-friendly but highly threshold-sensitive. ii) Gravity measures weight opportunities by impedance (time/cost), with Wilson’s entropy-maximizing framework giving them a rigorous foundation (2, 60). iii) Random-utility (RUM/logsum) measures define accessibility as expected utility in a behavioral model of location choice. Comparative typologies summarize these distinctions and their implications for practice (46, 61, 62).

Empirically, measurement choice (i.e., the choice between the three measures of access) alters the magnitudes and ranking of accessibility across distinct locations of a city. RUM often diverges from gravity measures where competition or access frictions constrain effective reach. In the Netherlands, job competition lowered effective access in dense cores under RUM, while gravity remained high (63). In Toronto, gravity overstated access where first- and last-mile barriers reduced reach, a gap captured by the RUM measure (64). In the United States, incorporating behavioral welfare through RUM (logsum) reordered policy rankings relative to gravity (65).

Cumulative and gravity measures were found to align when thresholds reflect typical commute times, but diverge elsewhere or by mode (66, 67). Designs that model demand–supply balance, such as floating catchment or hybrid approaches, shift neighborhood rankings yet again and alter appraisal footprints (68, 69). Cross-city evidence shows that whether centers or peripheries appear more equitable can change depending on the chosen indicator and threshold (40, 41). In short, measurement is not a technical afterthought: The choice between the cumulative, gravity, and RUM approaches fundamentally shapes conclusions about where transport needs are unmet, and who should be prioritized for transport interventions (see *SI Appendix*, section A and Table SI1 for a comparative evidence table).

Guided by this evidence, we pursue three empirical objectives. First, we compare the three principal families of accessibility measures at the neighborhood scale to show how measurement choice reshapes the map of transport inequality and, by extension, which communities are to be prioritized by transport policy. Second, we move beyond correlation to estimate the causal relationship between accessibility and deprivation, addressing confounding via a statistically robust instrumental variables (IV) based design that leverages road safety-based instruments. Third, we examine how effects vary across domains of deprivation and space, recognizing that equity impacts are multidimensional.

## London as a Test Case for Access and Deprivation

London provides a critical testbed for examining how accessibility relates to deprivation. Despite sustained investment in urban rail, including the Jubilee Line Extension, the Overground, and the Elizabeth Line, marked inequalities persist. The city offers detailed multimodal network data and fine-grained deprivation statistics, enabling high-resolution comparisons of accessibility measures and a credible identification strategy for causal inference.

London is the United Kingdom’s largest urban agglomeration (1,572 km^2^; 8.9 million residents; 5.3 million jobs in 2022), divided into 32 boroughs and 983 Middle Layer Super Output Areas (MSOAs). It is served by one of the world’s most extensive multimodal transport systems managed by Transport for London (TfL). Travel is highly multimodal: Public transport accounts for about 31% of daily trips, private vehicles 38%, walking 27%, and cycling 4.5% (70). This rich network coexists with pronounced socioeconomic divides, making London an ideal setting to explore how accessibility improvements interact with underlying patterns of exclusion and deprivation (16, 43, 44, 71–73).

In the broader literature, social exclusion has been used to describe the social and institutional processes that limit participation in the “customary life of society” (43). Originating in French policy debates on groups excluded from the welfare state and later adopted in the UK context (73), exclusion is often conceptualized spatially in transport studies: Unequal access to services and opportunities produces marginalization across the urban landscape (72). In practice, these processes are measured through deprivation, which reflects unmet needs and accumulated disadvantage across domains such as income, health, education, and housing (44, 71).

**Fig. 2.**
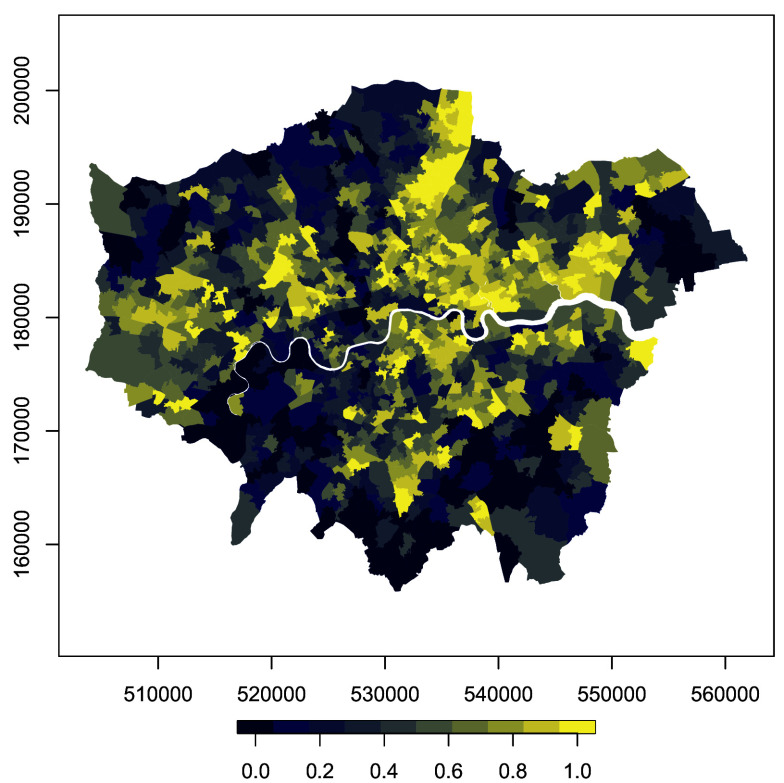
2019 weighted IMD for London MSOAs.

In England, the Index of Multiple Deprivation (IMD) operationalizes this concept, combining seven weighted components, income (22.5%), employment (22.5%), health, education (13.5%), crime (13.5%), housing (9.5%), and living environment (9.5%), to provide the most widely used small-area indicator of disadvantage and to guide resource allocation. For this study, we use the 2019 IMD scores, rebased for London and aggregated to the 2011 MSOA geography. Scores are normalized so that the most deprived MSOA takes a value of 1 and the least deprived takes 0. Fig. 2 maps the overall IMD, revealing marked spatial variation across the city. A full set of IMD maps by domain is included in *SI Appendix*, section F. Broadly, East London is more deprived than the West, though patterns differ by domain and exhibit substantial heterogeneity across boroughs and neighborhoods. This east–west divide holds for most domains, although the Living Environment domain stands out, with higher deprivation concentrated in central London.

While centered on London, our framework for contrasting accessibility measures and grounding equity claims in causal evidence is general and applicable to other metropolitan contexts.

## Accessibility Patterns Across London

**Fig. 3.**
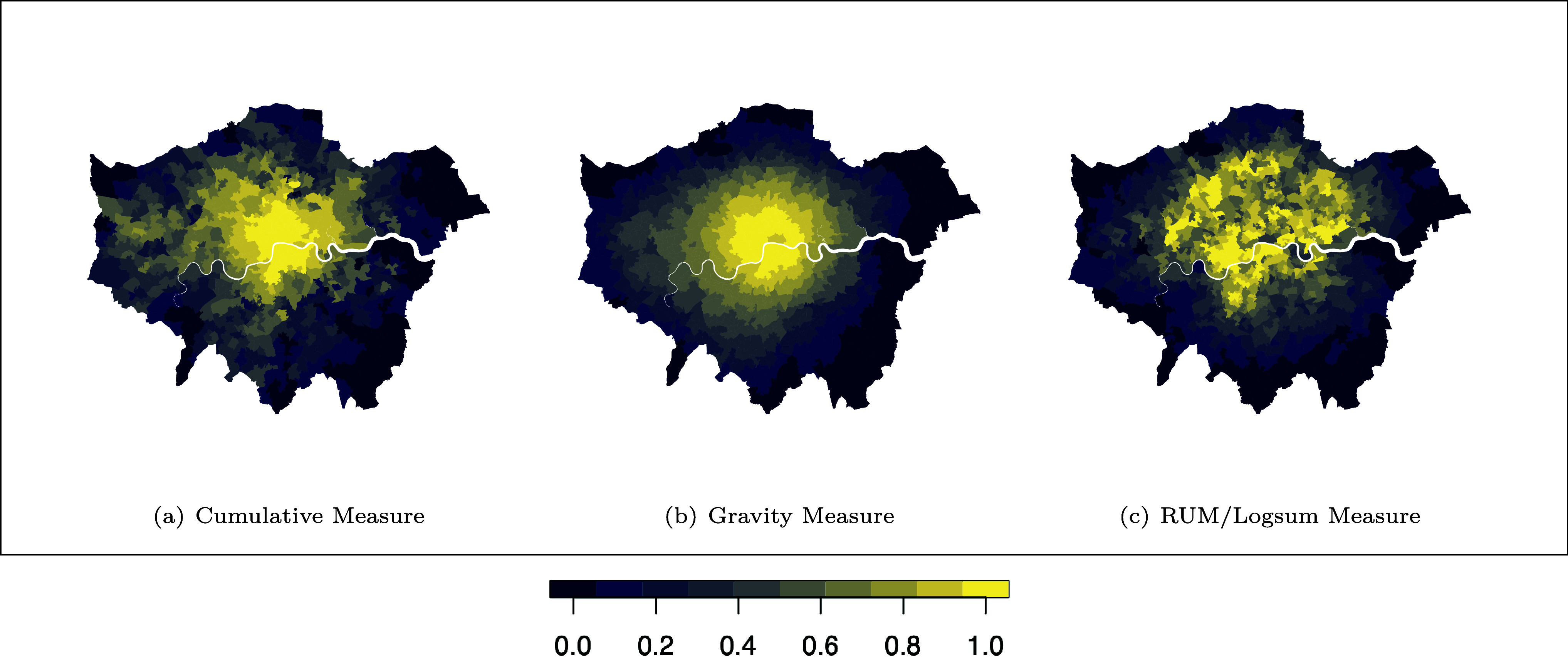
London accessibility measures [normalized to (0, 1)]. All three measures, (*A*) Cumulative Measure, (*B*) Gravity Measure, and (*C*) RUM/Logsum Measure, assign higher scores to central MSOAs and lower scores to peripheral MSOAs, but they differ in how sharply they differentiate neighborhoods.

We start by computing three standard accessibility measures for London. The formal definitions of these measures are provided in *SI Appendix*, section A and analytical decompositions of these measures are presented in *SI Appendix*, section B. *SI Appendix*, Fig. SI1 provides a conceptual overview of how each measure is constructed. Implementing these for London and normalizing each to [0,1] at the MSOA level, Fig. 3 shows that all three measures exhibit a central–peripheral gradient with higher scores in central London and lower scores toward the edge, while differing in how sharply they differentiate neighborhoods.

### The Cumulative (Isochrone) Measure.

In general form,

[1] $$\begin{array}{c} A_{\text{C}}^{\mathrm{ip}}=\sum_{j\in L_{\left(D|i\right)}^{p}}X_{j}^{p}, \end{array}$$

where $A^{\mathrm{ip}}$ is accessibility from origin *i* to opportunities of type *p*, $L_{\left(D|i\right)}^{p}$ is the set of destinations within a travel-time (or distance) threshold *D*, and $X_{j}^{p}$ is destination size. For this study, we use MSOA employment as $X_{j}^{p}$, include all MSOAs as destinations, and set D=45 min, reflecting established practice in London’s accessibility modeling (74, 75).

### The Gravity (Potential) Measure.

As first proposed in ref. 2, accessibility can be expressed as,

[2] $$\begin{array}{c} A_{\text{G}}^{\mathrm{ip}}=\sum_{j\in L^{\mathrm{ip}}}X_{j}^{p}\;f\left(c_{\mathrm{ij}}\right),\;f\left(c_{\mathrm{ij}}\right)=\;\exp\left(-\beta c_{\mathrm{ij}}\right), \end{array}$$

where $c_{\mathrm{ij}}$ is travel time or cost and *β* governs distance decay in a negative exponential decay function. Larger destinations contribute more, while farther or harder-to-reach destinations contribute less. Unlike cumulative measures, this approach avoids an arbitrary cut-off by continuously downweighting with impedance, and it is consistent with random-utility foundations under multinomial logit (76, 77). Reported *β* values vary by context, ranging from about 0.005 to 0.01 in car-based European studies to 0.2 to 0.3 for local service access (61, 78–82). In the absence of local calibration, we adopt β=0.01 as a conservative metropolitan-scale choice (83). We compute gravity-based accessibility across all MSOA pairs, using employment as $X_{j}^{p}$ and mode-share weighted travel times encompassing driving, public transport, cycling, and walking.

### The RUM (Logsum) Measure.

Random-utility theory treats accessibility as the expected maximum utility across feasible mode-destination options. With observable attributes $Z_{j}$ and parameters *β*,

[3] $$\begin{array}{c} A_{\text{R}}^{\mathrm{ip}}=\;\ln\;\left(\sum_{j\in L^{\mathrm{ip}}}e^{\beta Z_{j}}\right), \end{array}$$

so accessibility is derived directly from behavioral choice (84). In practice, this “bottom–up” approach uses stated or revealed preference data on travel behavior together with traveler characteristics. In this study, travel disutility is computed using travel-time coefficients from ref. 85, applied consistently across modes.

### Spatial Patterns of Accessibility.

As shown in Fig. 3, all three measures highlight a central–peripheral gradient. To examine agreement beyond visual inspection, we apply *k*-means clustering to the triplet of accessibility measure values for each MSOA. Figures for the *k*-means clustering results can be found in *SI Appendix*, section C and Fig. SI4. Four clusters were identified as the optimal solution, supported by both i) the elbow method, which evaluates reductions in within-cluster sum of squares, and ii) the silhouette method, which assesses the degree of separation between clusters. When mapped, they form near-concentric bands across London, indicating a systematic pattern in which the measures move together despite their different constructions. We combine the two inner clusters (C and D) for subsequent analysis, naming it Cluster CD, Cluster B is the intermediate band, while Cluster A is the peripheral band. Clustering, therefore, captures broad agreement in spatial structure. To probe remaining differences that matter for equity appraisal, we next turn to pairwise comparisons of the measures.

## Comparison of Accessibility Measures

While clustering reveals how the three measures move together, pairwise comparisons quantify the strength and form of their associations. We, therefore, examine each pair while allowing for potential nonlinearity. For every pairing, we report: i) Pearson’s correlation *r* representing the strength of linear association; ii) Spearman’s rank correlation *ρ* signifying the strength of monotonic association; and iii) model fits comparing a linear regression (with goodness-of-fit $R^{2}$) to a flexible spline (with effective degrees of freedom, EDF), using the change in Akaike Information Criterion, ΔAIC to test whether curvature improves fit, where negative ΔAIC favors the spline.

**Fig. 4.**
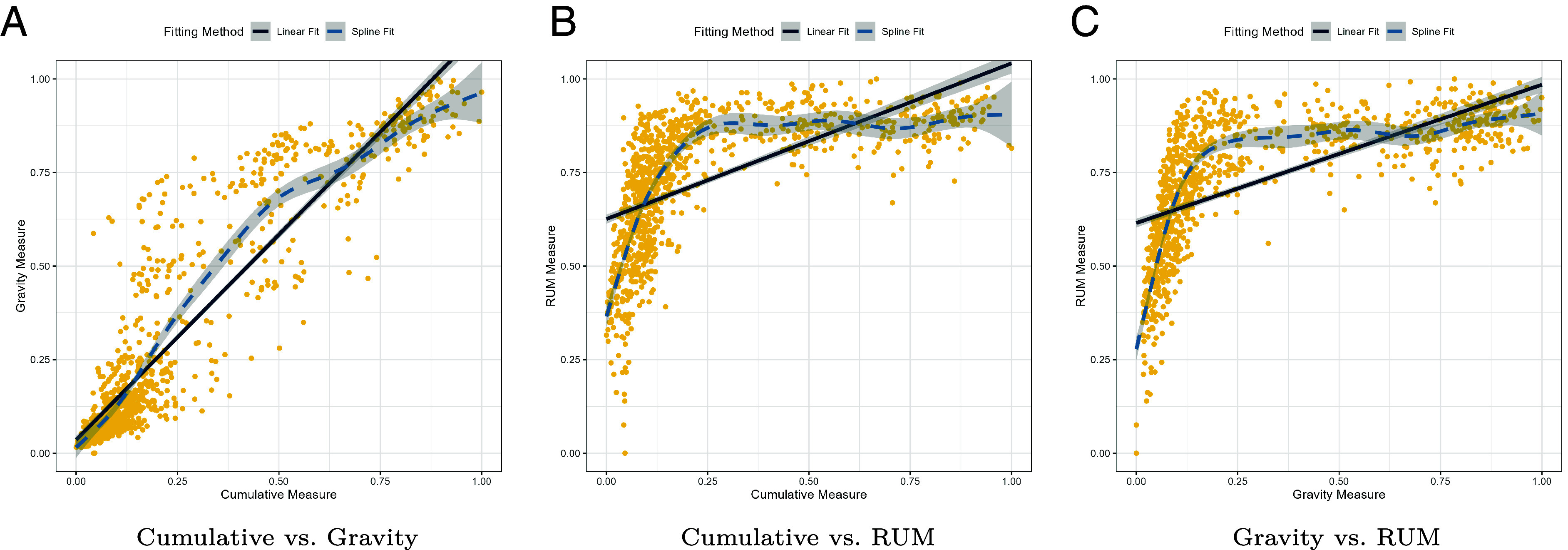
Nonlinear associations among accessibility measures. Points are MSOAs; lines show linear (solid) and spline (dashed) fits. Cumulative–Gravity is close to linear. Relationships with RUM are weaker in linear terms but strongly monotonic and notably nonlinear.

Fig. 4 visualizes these associations with linear (solid) and spline (dashed) fits, with each point in the figure corresponding to an MSOA. The first panel (Fig. 4*A*) shows a strong, near-linear relationship between the cumulative and gravity measures (Pearson r=0.916, Spearman ρ=0.899, linear $R^{2}=0.839$). A spline captures mild curvature (EDF = 4.83) and is favored by AIC (ΔAIC = −154.2), but the incremental gain over the high linear fit is limited, which is consistent with the visual impression of almost linear alignment.

By contrast, pairings with the RUM measure are monotonic but clearly nonlinear. For Gravity–RUM (Fig. 4*C*), the linear association is moderate (r=0.641, $R^{2}=0.411$), while rank agreement is strong (ρ=0.815); additional spline flexibility (EDF = 7.05) substantially improves the fit (ΔAIC = −738.4). The Cumulative–RUM comparison (Fig. 4*B*) shows a similar pattern (r=0.603, ρ=0.799, $R^{2}=0.363$, EDF = 6.08, ΔAIC = −581.5). Taken together, Table 1 and Fig. 4 indicate that all measures produce broadly consistent rankings of MSOAs (high *ρ*), but the RUM measure maps to the other measures in a strongly monotonic, nonlinear way. *SI Appendix*, Fig. SI5 maps the residuals from the spline fits. Residual maps indicate near-linear agreement between cumulative and gravity measures, but systematic spatial divergence for RUM due to compression of accessibility, especially in high-access, central regions.

**Table 1.** Strength and form of relationships between accessibility measures

Comparison	*r*	*ρ*	$R^{2}$	EDF	ΔAIC
Cumulative/Gravity	0.916	0.899	0.839	4.83	−154.2
Cumulative/RUM	0.603	0.799	0.363	6.08	−581.5
Gravity/RUM	0.641	0.815	0.411	7.05	−738.4

*Notes:r* = Pearson; *ρ* = Spearman; $R^{2}=$ linear model; EDF = spline complexity; and ΔAIC = AIC(spline)–AIC(linear).

It is worth emphasizing that these differences arise from the construct of the measures rather than from particular design choices in our implementation, for instance, parameter inputs for the RUM measure. This interpretation is consistent with the decomposition analyses in *SI Appendix*, section B, which show how each measure effectively ranks zones based on distinct conceptual ingredients.

## The Impact of Accessibility on Deprivation

Our goal is to estimate the effect of accessibility on deprivation, moving from associations and potentially spurious relationships to causal inference.

We begin with an ordinary least squares (OLS) model using the 2019 cross-section: The dependent variable is the Yeo–Johnson–transformed IMD (normalized to [0,1]), and the key explanatory variable in successive specifications is one of the three accessibility measures (cumulative, gravity, RUM), each also normalized to [0,1] (*Materials and Methods*). Table 2 reports results. The OLS coefficients are positive and statistically significant for all three measures, implying that more accessible places appear more deprived in this cross-section. This pattern is consistent with the colocation channel documented earlier with central areas scoring high on both access and deprivation and with sorting and legacy investment processes (Fig. 1).

**Table 2.** OLS and IV regression results: Accessibility measures and IMD

Accessibility measure	OLS	2SLS
Cumulative	1.017*** (0.110)	−1.297** (0.393)
Gravity	0.775*** (0.098)	−0.861*** (0.252)
RUM	1.794*** (0.184)	−3.234** (1.195)

*Notes:* Estimates are from OLS and IV (2SLS) regressions using the Junction Severity ratio as the instrument. First-stage F-statistics: Cumulative = 163.754, Gravity = 286.316, RUM = 49.557. Robust SEs in parentheses. ***P<0.001, **P<0.01, and *P<0.05.

### Addressing Confounding with Instrumental Variables.

**Fig. 5.**
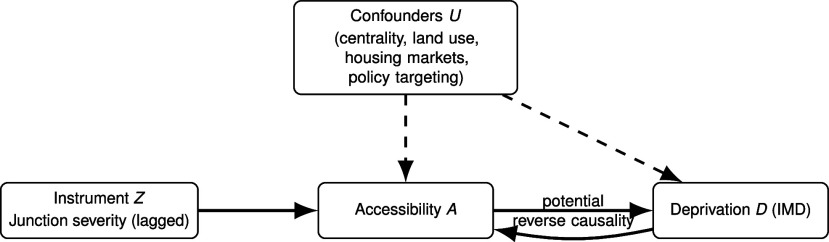
Directed acyclic graph (DAG). Unobserved context *U* confounds A→D. The instrument *Z* shifts *A* but is assumed not to affect *D* except through *A* (exclusion). The curved arrow indicates possible reverse causality from *D* to *A*.

Since accessibility and deprivation may both be shaped by unobserved contextual factors, such as the centrality of an area, the land-use mix, housing markets, and policy targeting (Fig. 5), OLS estimates can conflate correlation with causation. To address this, we use an instrumental-variables (IV) approach and introduce an instrument: the junction accident severity ratio (JSR), to separate cause from context in the relationship between accessibility and deprivation. The JSR is defined as the ratio of severe peak-hour crashes at junctions to all peak-hour crashes at junctions. For the IV strategy to work, the JSR must be a) related to accessibility or relevant but must b) influence deprivation only through its effect on accessibility or must satisfy the exclusion restriction.

Traffic safety research supports the claim that the JSR is relevant. Junctions account for a large share of crashes, and greater junction complexity, for example, multileg layouts, phasing, weaving, irregular geometry, increases crash severity (86–88). The JSR proxies for junction design complexity and complex, high-flow junctions where accidents occur are characteristic of highly accessible areas (89). To ensure that the JSR meets the exclusion restriction, we make deliberate design choices. For each MSOA we define a unique JSR that a) includes peak hour crashes only, so that the JSR reflects junction design characteristics rather than the effects of behavioral factors, such as drunk-driving or daily temporal factors, such as variability in enforcement of traffic safety rules during nonpeak hours b) uses time-lagged crash data, assuming that past junction conditions affect current accessibility but not current deprivation directly c) finally we aggregated the JSR within a 2 km radius of each MSOA centroid to smooth idiosyncratic local effects, such as traffic signal failures or unusually hazardous intersections, irrespective of junction design complexity. Aggregating at a higher level of analysis is common in empirical work, for example, refs. 90 and 91.

We also empirically test both relevance and exogeneity. The first stage F-statistics exceed the conventional threshold of 10 (163.754, 286.316, and 49.557; Table 2), confirming a strong first stage (92). To assess exogeneity, we use an alternative instrument restricted to crashes within the MSOA; standard overidentification tests (93) indicate that the instruments are jointly valid, supporting the exclusion restriction. Further construction details are provided in *Materials and Methods*, and additional discussion on the exclusion restrictions and limitations of the JSR are provided in *SI Appendix*, section D.

### From OLS to 2SLS: Overall Effects.

Two-stage least squares (2SLS) estimates (Table 2), which use the instrumental variable (IV) to isolate exogenous variation in accessibility, reverse the OLS sign: They suggest that higher accessibility leads to lower deprivation, ceteris paribus, that is, with all other contextual factors held constant. The estimated effects are statistically significant for all three measures, aligning with the “opportunity expansion” channel (Fig. 1) and indicating that the positive OLS associations likely reflect confounding, such as sorting, historical network concentration, and related features of *U*, in Fig. 5.

### Heterogeneity by IMD Domain: Multidimensional Deprivation.

Table 3 reports domain-specific results. For Income and Employment, OLS suggests that more accessible areas are more deprived; 2SLS reverses this to negative and significant coefficients across measures, consistent with accessibility reducing labor-market deprivation. For Education and Housing, OLS is weak or inconsistent, whereas 2SLS yields negative and significant effects, suggesting improved access is causally linked to better educational and housing conditions. For Health and Crime, OLS indicates positive correlations, however, 2SLS estimates are statistically insignificant, indicating that improvements in accessibility do not lead to higher deprivation across the two domains. For the Living Environment domain, both OLS and 2SLS are positive: Higher accessibility leads to greater environmental deprivation, consistent with environmental externalities in dense, highly connected areas. These domain results echo the theoretical channels in Fig. 1: Opportunity expansion dominates for income, employment, education, and housing, while context and externalities shape health, crime, and the living environment.

**Table 3.** OLS and IV results: Accessibility measures and IMD domain scores

Domain	Accessibility	OLS	2SLS
Income	Cumulative	0.823*** (0.121)	−1.409*** (0.384)
	Gravity	0.582*** (0.107)	−0.936*** (0.245)
	RUM	1.422*** (0.187)	−3.514** (1.180)
Employment	Cumulative	0.665*** (0.122)	−1.070** (0.365)
	Gravity	0.472*** (0.108)	−0.711** (0.236)
	RUM	1.016*** (0.186)	−2.668* (1.047)
Education	Cumulative	−0.224 (0.117)	−3.090*** (0.396)
	Gravity	−0.412*** (0.099)	−2.053*** (0.235)
	RUM	−0.341 (0.191)	−7.706*** (1.493)
Health	Cumulative	1.119*** (0.122)	−0.387 (0.346)
	Gravity	0.931*** (0.109)	−0.257 (0.227)
	RUM	1.406*** (0.181)	−0.965 (0.899)
Crime	Cumulative	1.123*** (0.131)	−0.062 (0.338)
	Gravity	0.920*** (0.108)	−0.041 (0.224)
	RUM	1.947*** (0.177)	−0.154 (0.850)
Housing	Cumulative	0.055 (0.106)	−3.256*** (0.474)
	Gravity	−0.099 (0.094)	−2.163*** (0.285)
	RUM	1.132*** (0.186)	−8.121*** (1.876)
Living Env.	Cumulative	1.965*** (0.104)	2.789*** (0.285)
	Gravity	1.863*** (0.083)	1.853*** (0.183)
	RUM	3.509*** (0.146)	6.955*** (0.885)

*Notes:* Estimates are from OLS and IV (2SLS) regressions using the Junction Severity Ratio as the instrument. First-stage F-statistics: Cumulative = 163.754, Gravity = 286.316, RUM = 49.557. Robust SEs in parentheses. ***P<0.001, **P<0.01, and *P<0.05.

### Impact of the Choice of Accessibility Measure.

In this section, we examine how the choice of accessibility measures impacts the analysis of the relationship between accessibility and deprivation. In other words, we examine the explanatory power of each measure, within the spatial patterns of compliance in accessibility measures identified in Spatial Patterns of Accessibility. We estimate models within the *k*-means clusters (*SI Appendix*, Fig. SI4): four near-concentric rings, with Cluster A outermost and Cluster D the core (C and D combined in estimation). Table 4 summarizes the results. In Cluster A (outer ring), IV estimates reveal a strong negative and statistically significant association between gravity-based accessibility and deprivation, while cumulative and RUM effects are imprecise and insignificant. In Cluster B (next ring), the IV estimate using gravity shows a negative and significant relationship, whereas both cumulative and RUM measures are imprecise and insignificant. In the inner clusters (C/D), the IV estimate using gravity retains a negative and significant association with deprivation, while cumulative is only weakly significant, and RUM remains highly unstable and insignificant. Overall, the gravity measure provides the most consistent and statistically significant negative effect across space, especially within intermediate and inner rings, whereas cumulative and RUM coefficients are less stable and often switch sign. This pattern indicates that although the three accessibility measures yield broadly coherent results in aggregate, as shown in Table 2, their behavior diverges across space, with gravity-based accessibility remaining the only measure that supports robust causal identification within clusters.

**Table 4.** Regression results by cluster: Accessibility and IMD

Cluster	Accessibility	OLS	IV (2SLS)
A	Cumulative	−1.046 (1.625)	−233.090 (463.965)
	Gravity	−1.219 (1.795)	−47.700* (20.798)
	RUM	0.693 (0.580)	−2,117.210 (131,616.106)
B	Cumulative	−0.279 (0.937)	200.774 (482.707)
	Gravity	0.507 (0.781)	−10.067*** (2.964)
	RUM	1.929** (0.638)	26.676 (14.405)
CD	Cumulative	1.070*** (0.217)	−8.710* (4.112)
	Gravity	0.689* (0.292)	−3.857*** (0.966)
	RUM	0.067 (0.982)	−164.831 (330.196)

*Notes:* OLS and IV (2SLS) estimates are reported for each specification. Robust SEs in parentheses. ***P<0.001, **P<0.01, and *P<0.05.

In sum, across models and scales, three conclusions emerge. i) Naïve cross-sectional correlations are misleading: OLS colocations reflect confounding by centrality, land use, and market forces. ii) Causal estimates indicate benefits: 2SLS results relate higher accessibility with lower deprivation overall and across key domains (income, employment, education, housing). iii) Measurement matters: The gravity measure yields the most stable signal across space; RUM’s nonlinear mapping and weaker first stage can shift local inferences, as shown in Table 4. These findings connect directly to our motivation: Both identification strategy (correlation versus causation) and measurement choice (cumulative/gravity/RUM) shape who appears to benefit and, thus, which areas are prioritized by transport interventions.

## Discussion

Our results speak to a central policy question: Does improving transport accessibility reduce deprivation, and can we trust the way we measure it? Two points follow in sequence. First, for describing London’s spatial distribution of access, three prominent measures (cumulative, gravity, and RUM/logsum) rank places similarly, with cumulative and gravity measures being especially aligned (*Accessibility Patterns Across London* and *Comparison of Accessibility Measures*). This corroborates earlier work, which reported strong correlations across functions and scales (66, 94). The alignment is not universal, however, as studies in other metropolitan contexts, such as in São Paulo, show departures between cumulative and gravity measures, cautioning against universal reliance on a single threshold–based measure (68). In short, simpler measures can be adequate for descriptive mapping of accessibility in London, but the choice of measure remains a first-order decision when generalizing beyond this setting.

Second, moving from associational to causal relationships changes the conclusion. Associational relationships obtained via ordinary least squares regressions suggest that high access colocates with high deprivation, measured via the Index of Multiple Deprivation, IMD, which is consistent with sorting and legacy investment patterns highlighted in the theoretical literature. However, once we address confounding using a causal statistical design based on instrumental variables estimation, the sign in the obtained causal relationship reverses: Higher accessibility leads to lower deprivation. Domain-level estimates align with a theoretical opportunity expansion channel for income, employment, education, and housing, while health and crime show no detectable effects. Further, deprivation in the living-environment domain is found to be causally linked with higher levels of deprivation. This pattern indicates that any equity gains from access improvements should be paired with strategies to manage local environmental costs.

Two methodological lessons follow. Measurement matters, said equivalently, which communities appear “priority” can change with the chosen accessibility measure, especially in dense cores and intermediate rings. In our data, gravity delivers the most stable negative relationship across spatial clusters, especially in the intermediate and inner rings, while cumulative and RUM produce statistically insignificant weaker or conflicting signals in specific bands consistent with their constructions and with the nonlinear cross-measure mappings we document. Causal identification matters; thus, we recommend presenting descriptive distributions (maps, inequality summaries) alongside a causal design. Doing both reduces the risk that policy priorities reflect threshold choices, decay functions, or confounded colocations rather than transport’s independent contribution to disadvantage.

The identification strategy is specific to the institutional and spatial features of our study area, and the causal effects reported here should therefore be interpreted as causal within this setting. Our study design is cross-sectional; while the IV strategy helps address confounding, it cannot recover dynamic effects or credibly account for reverse causality (for example, deprivation shaping subsequent network investment) by construction. *SI Appendix*, section D examines key threats to the validity of the JSR instrument, such as accident underreporting and targeted safety-related infrastructure investment in London, and argues that, while nontrivial, these issues are unlikely to compromise identification in our setting. A clear next step is to construct a panel of accessibility and IMD at an appropriate spatial scale and re-estimate the models presented here.

A second priority is to open the “black box” between higher accessibility and lower deprivation by measuring mechanisms. On the opportunity-expansion side, candidate mediators include labor-market reach (jobs within wage/skill bands and feasible travel budgets), time-to-care and time-to-school, childcare/elder care access, and indicators of civic and social participation. On the market-adjustment side, we recommend assembling annual data on rents, prices, tenure mix, turnover, and composition change; these would enable formal mediation tests of capitalization, gentrification/displacement, and relocation (including the possibility that lower-income households choose high-access areas to avoid the costs of auto-dependence) (7). Linking mechanism measures to our accessibility clusters (outer/intermediate/inner rings) would also reveal where affordability safeguards and environmental mitigation are most critical.

Finally, future work should integrate outcome-based and distributional perspectives on equity. Our approach connects access to material outcomes via IMD; this can be complemented by population-distribution summaries, such as Lorenz and Gini statistics (17, 95), Palma ratios (38, 96), and rank-size metrics (81), to reveal who holds the accessibility gains. On the supply side, comparing station density, service frequency, and temporal span against the geography of equity-seeking groups remains valuable (56). We therefore recommend reporting i) results across at least two conceptually distinct accessibility measures (cumulative, gravity, and, where possible, a behaviorally grounded logsum), ii) both associational and causal estimates, iii) domain-level and place-based heterogeneity, and iv) complementary supply- and opportunity-based indicators. See *SI Appendix*, section E and Fig. SI8 for Transport for London’s practice, on measuring supply side indicators, and the application of Lorenz Curves for all accessibility measures. Taken together, and consistent with international goals for inclusive, resilient, and sustainable cities (23), these steps would make accessibility evaluation more comparable across contexts and more actionable for directing investments to areas where access improvements most plausibly reduce multidimensional disadvantage while managing environmental externalities.

## Materials and Methods

### Pre-Processing the Data.

All accessibility indices and IMD scores were normalized via min-max scaling to the interval [0,1]:

[4] $$\begin{array}{c} x_{n}\;=\;\frac{x_{i}-\min\left(X\right)}{\max(X)-\min(X)}, \end{array}$$

where $x_{n}$ is the normalized value for MSOA *i*, $x_{i}$ is the raw value, and min(X) and max(X) denote the minimum and maximum across all MSOAs. Normalization enables comparison across the three accessibility indices within London and yields a relative interpretation: Higher $x_{n}$ indicates greater accessibility compared with other MSOAs, while for IMD, higher $x_{n}$ indicates greater deprivation.

To reduce skewness in the IMD distribution, we apply the Yeo-Johnson transformation (97), which accommodates both nonnegative and negative values. For an observation $x_{i}$ and parameter *λ*, the transformed value $x_{t}$ is

[5] $$\begin{array}{c} x_{t}=\left\{\begin{array}{cc} \frac{{\left(x_{i}+1\right)}^{\lambda }-1}{\lambda }, & x_{i}\geq 0,\;\lambda \neq 0,\\ \log\left(x_{i}+1\right),\; & x_{i}\geq 0,\;\lambda =0,\\ -\frac{{\left(-x_{i}+1\right)}^{2-\lambda }-1}{2-\lambda },\; & x_{i}<0,\;\lambda \neq 2,\\ -\log\left(-x_{i}+1\right),\; & x_{i}<0,\;\lambda =2. \end{array}\right. \end{array}$$

The parameter *λ* is estimated, for instance, by maximum likelihood to best approximate normality. This transformation improves model fit by reducing skewness and downweighting the influence of extreme values.

### Causal Inference Framework.

#### Model specification and estimation.

We aim to estimate the causal impact of accessibility on deprivation. Let $A_{i}\in \left[0,1\right]$ denote an accessibility index for spatial unit (MSOA) *i* (cumulative, gravity, or RUM/logsum; normalized), and let $Y_{i}\in \left[0,1\right]$ denote the IMD outcome (Yeo-Johnson-transformed and normalized). We model a dose–response relationship:

[6] $$\begin{array}{c} Y_{i}\;=\;\alpha \;+\;\beta \;A_{i}\;+\;\varepsilon_{i}, \end{array}$$

where $\varepsilon_{i}$ is a mean-zero disturbance. As a baseline, we estimate Eq. **6** by ordinary least squares estimation (OLS).

As discussed in *The Impact of Accessibility on Deprivation*, OLS is vulnerable to confounding: Unobserved context $U_{i}$ (for instance, centrality, land-use mix, housing markets, policy targeting) may jointly influence the treatment dose $A_{i}$ and the response $Y_{i}$, and reverse causality (deprivation shaping subsequent network investment) is plausible (refer to Fig. 5). To address this, we adopt instrumental variables (IV) estimation and obtain coefficients via two-stage least squares (2SLS) using an appropriate instrument $Z_{i}$:

[7] $$\begin{array}{cc} \text{First stage:}\; & A_{i}\;=\;\pi_{0}\;+\;\pi_{1}Z_{i}\;+\;\nu_{i}, \end{array}$$

[8] $$\begin{array}{cc} \text{Second stage:}\; & Y_{i}\;=\;\alpha \;+\;\beta \;{\hat{A}}_{i}\;+\;u_{i}. \end{array}$$

The instrument must satisfy: i) relevance ($\pi_{1}\neq 0$; empirically supported by strong first-stage *F*-statistics reported in Table 2, following 92); ii) exclusion (the instrument shifts $Y_{i}$ only through $A_{i}$) and exogeneity (independence of $Z_{i}$ from unobserved determinants of $Y_{i}$). Under these conditions, 2SLS identifies the causal coefficient *β* in Eq. **6**.

#### Potential outcomes interpretation.

We place this strategy within the potential-outcomes framework for causal inference. Let $Y_{i}\left(a\right)$ denote the deprivation that would be observed for MSOA *i* if its accessibility were set to *a*. The average potential outcome (APO), or average dose–response function, is

$$m\left(a\right)\;=\;E[\;Y_{i}\left(a\right)\;].$$

Our target is the slope of m(a), that is, the average causal response to marginal changes in accessibility. If the structural relation is linear in *a*,

m(a) = α + βa,

then *β* is the (constant) causal dose–response slope. OLS identifies *β* only under three standard conditions: i) unconfoundedness ($Y_{i}\left(a\right)\;⊥⊥\;A_{i}$ for all *a*), ii) common support/positivity (each value of *A* under consideration occurs with positive probability across MSOAs), and iii) SUTVA (well-defined treatment and no interference across units). In our context, condition i) is implausible because unobserved context $U_{i}$ (centrality, land-use mix, housing markets, policy targeting) likely affects both $A_{i}$ and $Y_{i}$; ii) is reasonably supported by the broad empirical range of $A_{i}$ across MSOAs; and iii) is an approximation in spatial settings, given potential spillovers along networks. We ensure the validity of SUTVA by choosing the spatial units to be sufficiently large. Consequently, OLS in Eq. **6** is treated as associational.

IV replaces unconfoundedness with the relevance and exclusion and independence conditions on $Z_{i}$. Under these, 2SLS estimates a local causal effect: The average derivative of m(a) for MSOAs whose accessibility is shifted by $Z_{i}$ (a “complier” interpretation; see ref. 98). With linear effects, this local effect coincides with *β*, the slope of the APO. The DAG in Fig. 5 encodes these assumptions: Unobserved context *U* confounds A→Y; the instrument *Z* shifts *A* but has no direct path to *Y*; a red curved arrow marks potential reverse causality from *Y* to *A* that the exclusion assumption are intended to rule out.

In practice, we estimate Eq. **6** and Eqs. **7** and **8** separately for each accessibility index, with $A_{i}$ and $Y_{i}$ normalized to [0,1] and $Y_{i}$ Yeo-Johnson-transformed to reduce skewness (Pre-Processing the Data). OLS coefficients are interpreted as associational slopes; 2SLS coefficients are interpreted as causal local slopes of the average dose–response among MSOAs whose accessibility is moved by the instrument.

### Instrumental Variable.

We derive a valid external instrument from traffic casualty data, adapting a recent instrument from the agglomeration literature (see ref. 99).

**Fig. 6.**
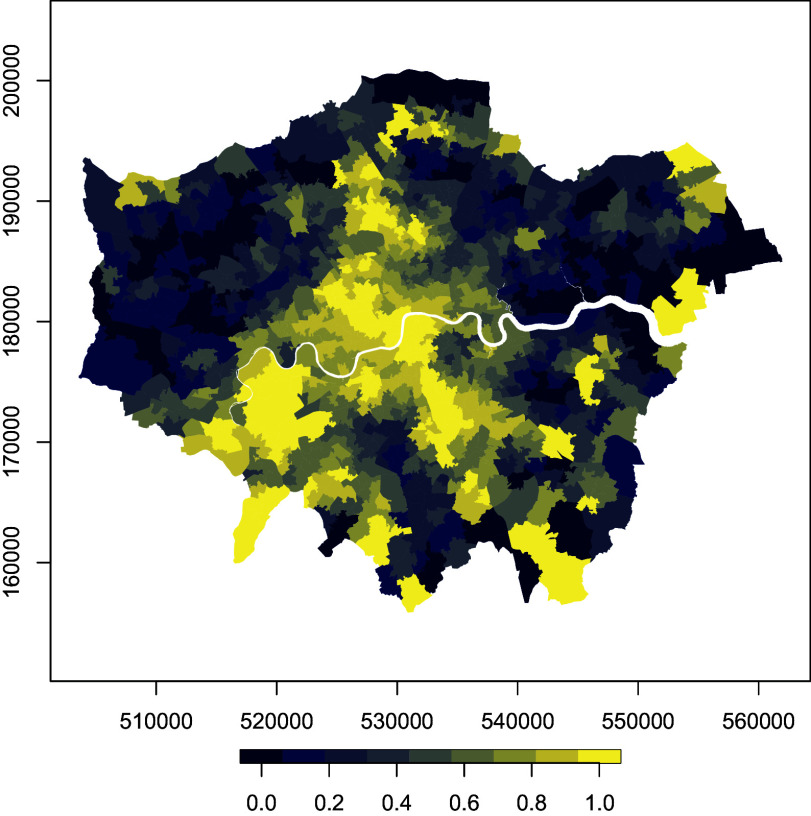
Junction severity ratio, 2 KM radius.

We define the Junction Severity Ratio (JSR) at the MSOA level using a floating catchment area (FCA) approach. For each MSOA *i*, the JSR is defined as

$${\text{JSR}}_{i}\;=\;\frac{S_{i}^{\left(2\;\mathrm{km}\right)}}{A_{i}^{\left(2\;\mathrm{km}\right)}},$$

where $S_{i}^{\left(2\;\mathrm{km}\right)}$ denotes the total number of severe accidents at junctions occurring within a 2 km radius of the centroid of MSOA *i*, and $A_{i}^{\left(2\;\mathrm{km}\right)}$ denotes the total number of all accidents at junctions within the same catchment. Accident counts are pooled over the period 2005–2014 and are restricted to morning and evening peak periods. The 2 km catchment radius is chosen to balance local relevance with spatial smoothing. It is sufficiently large to average over highly localized infrastructure features and behavioral idiosyncrasies at individual junctions, while remaining small enough to capture the immediate road environment relevant to each MSOA (Fig. 6). Details on this can be found in *SI Appendix*, section D.

As discussed in *The Impact of Accessibility on Deprivation*, two conditions must be met for the instrument to be valid in this study: relevance (JSR is related to transport accessibility) and exogeneity (JSR is unrelated to deprivation through any channel other than transport accessibility).

Relevance is theoretically supported by traffic safety research showing that greater junction complexity increases accident severity by raising driver cognitive load (87, 88). In the London case study, dense urban areas also tend to feature older and more irregular road layouts than suburban radial corridors (100), contributing to higher JSR in high access zones.

Exogeneity is more difficult to establish directly, but the construction of the JSR supports this assumption. The instrument is time-lagged, covering accidents between 2005 and 2014, while deprivation is measured using the 2019 IMD; this temporal separation supports the exclusion restriction by limiting any direct influence of past accidents on current deprivation outcomes. The JSR is aggregated using a 2 km floating catchment area around each MSOA centroid; this reflects the wide variation in London MSOA sizes and provides a neighborhood-scale measure that smooths the influence of particularly dangerous junctions while remaining local and avoiding the broader spatial aggregation implied by larger catchments. By construction, the JSR reflects road-network complexity, which should not directly determine where deprivation is observed. Severe traffic accidents are relatively rare events and therefore unlikely to systematically affect house prices or perceptions of neighborhood safety in ways that would confound deprivation. To reduce behavioral confounding, the JSR includes only accidents occurring during peak hours, limiting the influence of crashes related to substance abuse or crime. As ratio of serious to total accidents, the JSR does not mechanically scale with traffic volumes, improving the plausibility of exogeneity, as traffic flows are related to local economic activity and deprivation. To further assess exogeneity, we constructed a complementary instrumental variable restricting the JSR to accidents within the MSOA only. The MSOA-level instrument is weakly correlated with the FCA-based measure (sample correlation = 0.17 and adjusted $R^{2}=0.1608$), indicating limited shared variation and mitigating concerns about multicollinearity. We conduct the Sargan test under classical SEs and the Hansen *J* test under heteroskedasticity-robust SEs (93, 101), relying on the latter to assess joint instrument validity in line with the main results. An in-depth discussion on the exclusion criteria and limitations of the JSR can be found in *SI Appendix*, section D.

In urban economics, instruments based on historical or structural transport features are widely used to study short-run economic outcomes such as productivity and house prices. Examples include historical populations (102–104), historic transport networks (25, 105, 106), and natural features such as soil suitability for tall buildings (105, 107).

We also tested historical population as an instrument, drawing on lagged population data from ref. 108. However, this variable did not meet the exogeneity criterion, since the spatial distribution of historic populations may have influenced the placement of infrastructure, which in turn could shape present-day deprivation patterns.

### Materials.

#### Travel time matrix.

The travel time matrix (TTM) used in this study was developed for each Middle Layer Super Output Area (MSOA) in Greater London (983 MSOAs in total) for four modes: driving, cycling, walking, and public transport (PT). Travel times were generated using the r5r package, which supports the generation of multimodal travel times.

#### Geographies.

The travel time matrices were generated for each MSOA for the morning peak only, using the population-weighted centroid (PWC) of each MSOA to represent both origins and destinations. The MSOAs correspond to the 2011 Census definitions.

#### Road network and timetables data.

An OpenStreetMap (OSM) extract was used to represent the road and pedestrian networks. For public transport, two sets of timetables were required: one for the bus network and one for the rail network. Bus data were sourced from the Bus Open Data Service (BODS). Rail data were obtained from the Rail Delivery Group and include both rapid transit and passenger rail in Greater London. Both timetable inputs were provided in General Transit Feed Specification (GTFS) format.

#### Travel time parameters.

For walking and cycling, the main input was the OSM network. Walking speed was set to 4.32 km/h (109), and cycling speed was set to 16 km/h, following Journey Time Statistics produced by the Department for Transport (DfT). For cycling, a “stress level” (1 = minimum, 4 = maximum) represents cyclists’ tolerance for traffic; this was set to two to represent the average cyclist. The maximum travel time for walking and cycling was 120 min.

For public transport, the main inputs were the bus and rail timetables. Travel times were generated assuming walking access and egress, with PT as the main mode. The maximum access/egress time was set to 15 min, the maximum number of transfers to 4, and the maximum total travel time to 120 min. For driving, the main input was OSM; link speeds were assumed to be the posted speed limits of the road segments as defined in OSM, with a maximum travel time of 120 min.

The departure time was set to 08:00 on 23 September 2024 for all modes. A 30-min departure window was used, with the routing engine computing one travel time per minute within the window. The 50th percentile of travel times for each mode was used in the analysis; where the 50th percentile lay outside the specified maximum travel-time threshold for a mode, the 25th percentile was used.

#### Opportunities or measures of mass.

Employment data and population density were used as “opportunities” for the cumulative-count measures and as “masses” for the gravity measures. Employment data were taken from the Business Register and Employment Survey (BRES) for 2022 at the MSOA level (2011 definitions).

#### Mode shares.

Trip data from an anonymized version of the London Travel Demand Survey (LTDS) for 2022/23 were used to create mode shares for origin–destination pairs in this study. Mode shares were available at the borough level; the borough-level origin–destination mode shares were applied at the MSOA level.

#### Index of multiple deprivation.

The Index of Multiple Deprivation (IMD) is a ranked indicator of relative deprivation for small areas at the Lower Layer Super Output Area (LSOA) level in England. It is calculated by scoring LSOAs on 39 component indicators across seven domains, weighted as follows: a) income (22.5%), b) employment (22.5%), c) education, skills, and training (13.5%), d) health deprivation and disability (13.5%), e) crime (9.3%), f) barriers to housing and services (9.3%), and g) living environment (9.3%). While these indicators are produced at the LSOA level, they were aggregated to MSOAs using a population-weighted methodology, as recommended in the IMD technical guidance (110). The Income domain captures factors such as low household income and reliance on income-related benefits; the Employment domain reflects involuntary exclusion from the labor market due to unemployment, ill health, or disability; the Education domain includes measures of adult skills, school attainment, and attendance; the Health domain includes indicators such as morbidity, mental health, and years of potential life lost; the Housing domain reflects barriers such as affordability, overcrowding, and access to services; the Crime domain captures risks of personal and property crime; and the Living Environment domain covers indicators of housing quality and outdoor environmental quality (e.g., air pollution), implying that better-connected areas may be more exposed to environmental stressors.

#### Road safety data.

The Road Safety Data maintained by the UK Department for Transport derive from the STATS19 system; standardized police forms used to record all personal; injury road collisions in Great Britain since 1979. STATS19 provides linked collision, vehicle, and casualty records, including a unique collision ID (“accident index”), date/time, location and weather, road characteristics, vehicle details, casualty demographics and injury severity, plus up to six contributory factors. Police forces collect the data through CRASH (Collision Reporting And SHaring); DfT then processes and validates the dataset. The unique IDs and rich descriptors recorded for each crash to identify junction collisions and compute the Junction Severity Ratio (JSR). While the STATS19 data undergo extensive validation and verification procedures, underreporting, particularly for less severe collisions remains a recognized limitation. The STATS19 data may be incomplete due to a) personal injury collisions not reported to the police b) collisions reported to the police but STATS19 system was not used c) reported to the police and recorded using the STATS19 system, but with errors leading to exclusion of collision record.

## Supplementary Material

Appendix 01 (PDF)

## Data Availability

All data used in this study are publicly available from open data sources, including UK government datasets such as the English Indices of Deprivation (111), the Business Register and Employment Survey (112), STATS19 (113) road safety data, the Bus Open Data Service (114), and Rail Delivery Group timetable data (115), as well as OpenStreetMap data (116), with full access details provided in the references.
