# Supplementary material for: Uncovering how transport access reduces deprivation: When colocation misleads
Source: Proc Natl Acad Sci U S A. 2026 Apr 28;123(18):e2532730123. doi: 10.1073/pnas.2532730123 (PMC13142980; doi:10.1073/pnas.2532730123)
Supplement: Supplementary file 1 — Appendix 01 (PDF) [file pnas.2532730123.sapp.pdf]

PNAS

Supporting Information for

Uncovering How Transport Access Reduces Deprivation: When Co-location Misleads

Surabhi Ojha, Anupriya Anupriya, Daniel Hörcher, Daniel J. Graham

Correspondence to: d.j.graham@imperial.co.uk

This pdf includes:

- **SI Appendix A Transport Accessibility Defined**: In-depth review of definitions of the three transport accessibility measures used for analysis in this paper.
  - Fig. SI1: Conceptual diagram of the three transport accessibility measures.
  - Table SI1: Summary of the literature comparing the three accessibility measures.
- **SI Appendix B Decomposition of Transport Accessibility Measures**: Decomposition of each accessibility measure, examining how modelling choices affect results.
  - Fig. SI3: Effects of the decay parameter.
  - Fig. SI2: Effects of the travel time threshold.
- **SI Appendix C Comparing Accessibility**: Results from the k-means clustering exercise and residuals of pairwise comparisons between accessibility measures.
  - Fig. SI4: Results of the k-means clustering.
  - Fig. SI5: Residuals from pairwise comparisons of accessibility measures.
- **SI Appendix D Instrument Assumptions and Robustness Tests**: This supplement details the construction of the instrument, the Junction Severity Ratio (JSR), and robustness checks.
  - Fig. SI6: Visual comparison of JSR and the Index of Multiple Deprivation (IMD).
  - Fig. SI7: Comparison of JSR across aggregation radii.
  - Table SI2: Sensitivity test results at different radii.
  - Table SI3: Domain-level Sensitivity test results (set 1).
  - Table SI4: Domain-level Sensitivity test results (set 2).
- **SI Appendix E State of Practice in London**: Overview of London's practice in quantifying transport accessibility, including Public Transport Accessibility Level (PTAL).
  - Fig. SI8: Lorenz curve comparing accessibility metrics and PTAL.
- **SI Appendix F IMD by Domain**: Definition of the Index of Multiple Deprivation (IMD).
  - Fig. SI9: Maps of IMD scores by domain.

SI Appendix A Transport Accessibility Defined

Assumptions in the Construction of Accessibility Measures

The transport economics literature identifies four "axioms" of accessibility (Miller, 2019) that apply across measures: accessibility is (A) place-specific; (B) trip–purpose-specific; (C) dependent on the ease of travel and location attractiveness; and (D) dependent on the choice of the set of locations included in the calculation. Accessibility measures are built from choices made about four "elements": (A) travel disutility or impedance; (B) location attractiveness; (C) the role of individual tastes, preferences, and constraints in determining both travel impedance and location attractiveness; and (D) the set of locations to include in a given calculation (Miller, 2020).

We evaluate three measures of transport accessibility, cumulative count, gravity and RUM/logsum that are used in this research and discuss the choices for these four elements under each measure. In what follows, *study area* denotes a selected geography (e.g., a city or a country), and *zones* are lower-level subdivisions within the study area. We use *origin* and *destination* for selected zones within the study area.

Cumulative Count or Isochrone Measures

"Cumulative count" or "isochrone" measures sum the number of "places" or "opportunities" within a chosen distance or travel-time threshold. The general form is:

$$A^{ip} = \sum_{j \in L^p_{(D|i)}} X^p_j. \tag{SI1}$$

Here, A^{ip} is accessibility for origin zone i to locations of type p; $L^p_{(D|i)}$ is the set of locations of type p within the threshold D from origin i; and X^p_j measures the size of destination j of type p. Cumulative measures are widely used in transport planning and are also referred to as "points of interest" measures.

For cumulative measures, *travel disutility* may be represented by Euclidean distance, travel time, travel distance, or generalised cost. *Location attractiveness* is represented by counts of selected destination types (for example, schools, hospitals, jobs, population). The *role of individual tastes* can be reflected through the choice of the *set of locations* included (for example, defining school access for ages 12–18).

A limitation is that the choice of threshold D is arbitrary; the measure assumes travellers are indifferent among all *places* within D. Edge cases are handled poorly (for example, a site at 1.1 km is excluded when $D = 1$ km). Another limitation is that cumulative measures are typically defined for a single mode and are not prescriptive about combining multiple modes and their travel times. Finally, these measures are sensitive to the spatial distribution of the mass variable. Advantages include ease of computation and interpretation: the numerical value simply aggregates destination attractiveness within D, and values can be used to rank zones by relative accessibility.

Gravity Measures

The gravity measure, first introduced in (Hansen, 1959), improves on cumulative measures by (a) drawing locations from a *choice set* and (b) replacing a hard threshold with a continuous *impedance* function:

$$A^{ip} = \sum_{j \in L^{ip}} X^p_j \, f(d_{ij}), \tag{SI2}$$

where A^{ip} depends on destination size X^p_j and an impedance function $f(d_{ij})$ of distance or travel time, with $\frac{\partial f}{\partial d_{ij}} < 0$.

As with cumulative measures, *travel disutility* can be quantified using Euclidean distance, travel time, distance, or generalised cost; *location attractiveness* is represented by mass measures such as employment or population; and individual preferences can be incorporated by defining a group-specific *choice set*.

Gravity models, inspired by Newton's law of gravitation, are widely used in trade economics (Head and Mayer, 2014; Bergstrand, 1985), geography, and travel-demand modelling. They have also been linked to information theory (Shannon, 1948), which quantifies uncertainty in systems, and the principle of entropy maximisation has been used to derive such models by identifying the most probable spatial distribution of flows under given constraints (Wilson, 1971).

Critiques note that standard gravity models do not explicitly account for *competition* either in the mass measure (for example, job competition in high-employment zones) or across modes. Extensions address competition (for example, the Shen index) and multi-modality by, for example, using weighted travel times or the least-cost mode between zones (Shen, 1998). Gravity measures are relatively stable with respect to changes in mass and impedance specifications. While absolute gravity values lack a direct interpretation, *relative ranks* across zones provide a meaningful basis for comparison.

Random Utility Measures (RUM)

Random-utility–based measures (RUM) derive from neoclassical microeconomic theory. A "rational" traveller evaluates a feasible set of alternatives and chooses the option with the highest utility. The most prominently used RUM model is the multinomial logit (MNL). The location-choice probability is:

$$P_j^{ip} = \frac{e^{V_j}}{\sum_{j' \in L^{ip}} e^{V_{j'}}} = \frac{e^{\beta Z_j}}{\sum_{j' \in L^{ip}} e^{\beta Z_{j'}}}, \tag{SI3}$$

where $V_j = \beta Z_j$ is the systematic utility of alternative j, Z_j is a vector of explanatory variables (e.g., time, cost, service), and β is a vector of parameters.

Perceived utility is

$$U_j = V_j + \epsilon_j, \tag{SI4}$$

with ϵ_j capturing idiosyncratic tastes. The decision maker chooses the option that maximises U_j. While U_j is not observed, the expected maximum utility,

$$I^{ip} = \mathbb{E}\big[\max_j U_j\big] = \ln\left(\sum_{j \in L^{ip}} e^{\beta Z_j}\right), \tag{SI5}$$

is the *logsum*, which also serves as the associated accessibility measure:

$$A^{ip} = \ln\left(\sum_{j \in L^{ip}} e^{\beta Z_j}\right). \tag{SI6}$$

Under standard assumptions, the denominator in (SI3) relates to consumer surplus via the *logsum interpretation* (Ben-Akiva and Lerman, 1985).

In RUM measures, *travel disutility* is typically a generalised cost; *location attractiveness* enters through Z_j; and models can be tailored to specific trip types (e.g., business vs. leisure) or traveller characteristics (e.g., age, gender). Critiques focus on modelling and data demands: RUM measures require granular, high-quality survey or OD-flow data; utility is often specified at the zonal level (implying homogeneity of opportunities within zones); and utility functions may need to aggregate multiple destination attributes. Consequently, RUM measures are context dependent. Absolute accessibility values are not directly interpretable; their main utility is to produce *ordinal rankings* of places.

A conceptual illustration of these three measures is provided in Figure SI1.

Mathematical Equivalence of Gravity and RUM

Under specific functional forms, gravity measures and the RUM (logsum) measure are mathematically equivalent (Anas, 1983), yielding identical parameter estimates when applied to the same base data (Anas, 1983). A formal proof is provided in (Miller, 2020); here, we outline the equivalence, beginning with the logit destination-choice model.

Consider the gravity-based location-choice formulation:

$$P_j^{ip} = \frac{X_j^p \, f(d_{ij})}{\sum_{j' \in L^{ip}} X_{j'}^p \, f(d_{ij'})}. \tag{SI7}$$

Interpreting the numerator as an exponential of a systematic utility yields

$$P_j^{ip} = \frac{\exp\big(\ln X_j^p + \gamma d_{ij}\big)}{\sum_{j' \in L^{ip}} \exp\big(\ln X_{j'}^p + \gamma d_{ij'}\big)}, \tag{SI8}$$

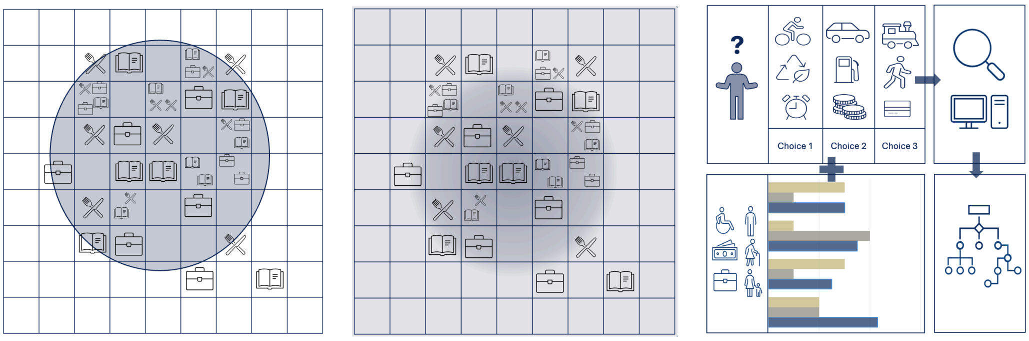

SI1(a) Cumulative Count Measure SI1(b) Gravity Measure SI1(c) RUM Measure

Figure SI1: Conceptual illustration of three approaches to measuring accessibility. (a) Cumulative: total opportunities within a fixed travel-time threshold. (b) Gravity: opportunities weighted by distance/time impedance. (c) RUM/logsum: accessibility as expected maximum utility over modes and destinations.

so that $V_j = \ln X_j^p + \gamma d_{ij}$. If we specify $f(d_{ij}) = \exp(\gamma d_{ij})$, (SI7) becomes

$$P_j^{ip} = \frac{X_j^p \, e^{\gamma d_{ij}}}{\sum_{j' \in L^{ip}} X_{j'}^p \, e^{\gamma d_{ij'}}} = \frac{\exp\big(\ln X_j^p + \gamma d_{ij}\big)}{\sum_{j' \in L^{ip}} \exp\big(\ln X_{j'}^p + \gamma d_{ij'}\big)}. \quad \text{(SI9)}$$

Since $X_j^p = \exp(\ln X_j^p)$, (SI9) is identical to (SI8), which matches the logit form in (SI3). Hence, the gravity formulation maps to the RUM/MNL structure with a particular specification of systematic utility, establishing the equivalence.

Comparative Evidence on Accessibility Measures

The table below summarises key studies comparing cumulative, gravity, and utility-based (RUM/logsum) accessibility measures across varied contexts, highlighting where methodological choices lead to differing conclusions.

Table SI1: Key literature highlighting disagreements between accessibility measures

**Paper**	**Geography**	**Measures Compared**	**Conclusions**
Geurs and Ritsema van Eck (2006)	Netherlands	Gravity vs. Utility Based	Utility-based accessibility diverged from potential measures: job competition lowered accessibility in dense cores, while gravity-based measures showed high accessibility.
Dong et al. (2006)	United States	Gravity vs. RUM (logsum)	Gravity ignores behavioural choice; RUM-based logsum produced welfare-consistent outcomes and changed the ranking of policy alternatives.
Hasnine et al. (2019)	Toronto, Canada	Gravity vs. RUM (logsum)	Gravity overestimated access where first-/last-mile barriers exist; RUM revealed significantly lower effective transit accessibility.
Kapatsila et al. (2023)	Eight Canadian Metros	Cumulative vs. Gravity	Found strong correlations between measures when thresholds match average commute times; cumulative may substitute for gravity in some contexts.
Giannotti et al. (2021)	São Paulo & London	Cumulative, Gravity, Floating Catchment Area	Rankings of neighbourhood accessibility varied by metric; cumulative overstated some areas, while floating catchment area better reflected demand–supply balance.
Klar et al. (2023)	Canada (Vancouver, Edmonton)	Cumulative, Gravity, Hybrid	Project appraisal differed across metrics: cumulative showed benefits in some areas that gravity and hybrid approaches did not, altering spatial extent of impacts.
Palacios (2022)	Montréal, Canada	Cumulative vs. Gravity	Found high correlation between measures when thresholds matched mean commute times, but results diverged at other thresholds or by mode.

SI Appendix B Decomposition of Transport Accessibility Measures

Decomposition of Cumulative Count Measures

We introduce the general form of a cumulative count measure:

$$A^{ip} = \sum_{j \in L^p_{(D|i)}} X^p_j. \tag{SI10}$$

From (SI10) there are two drivers of this measure: the *travel-time threshold* (D) and the definition of the *opportunity* (X^p_j). Cumulative count measures quantify accessibility by summing the opportunities surrounding origin i that fall within the threshold D.

Travel-Time Thresholds

As the threshold increases, accessibility values rise. At the extreme, if D is sufficiently large to connect all origins to all destinations, accessibility becomes uniform across zones. Larger thresholds therefore reduce spatial differentiation in accessibility. Figure SI2 illustrates this: accessibility values are based on employment as the mass measure and show density plots for cumulative accessibility using thresholds in 15-minute increments between 15 and 60 minutes. The choice of D is thus a critical determinant of the cumulative measure.

Mass Measures

Mass measures affect accessibility in a *localised* way: only destinations within the threshold contribute. For example, increasing employment at a destination raises accessibility values for origins that can reach it within D, but has no effect beyond that catchment.

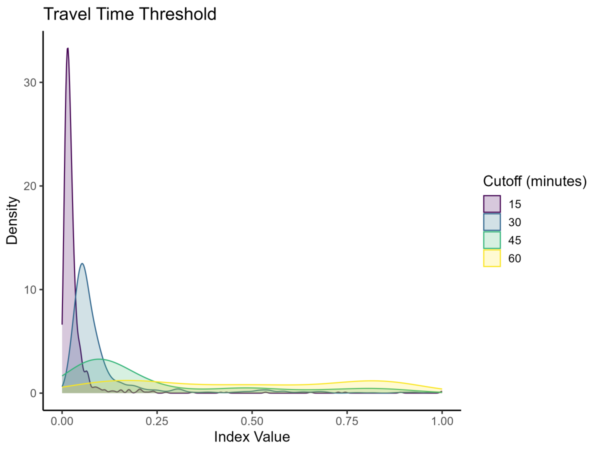

Figure SI2: Decomposition of cumulative count measures.

Decomposition of the Gravity Measure

Following the approach in (Graham and Gibbons, 2019), we decompose the gravity measure to clarify what it represents. The gravity index takes the form

$$A^{ip} = \sum_{j \in L^{ip}} X^p_j \, f(d_{ij}), \tag{SI11}$$

where X^p_j is the opportunity at destination j and $f(d_{ij})$ is an impedance function of distance (or generalised cost).

Applying the law of large numbers to A^{ip} yields

$$A^{ip} \xrightarrow{p} \mathbb{E}(X^p_j)\,\mathbb{E}\big(f(d_{ij})\big) + \mathrm{Cov}\big(X^p_j, f(d_{ij})\big). \tag{SI12}$$

The first term is constant across zones, capturing the mean opportunity level and mean impedance. The second term reflects the covariance between opportunities and impedance, and drives variation across zones. This variation arises from:

1. **Centrality:** For equally sized zones, central zones have lower mean distances to other zones than peripheral zones, raising $\mathbb{E}\big(f(d_{ij})\big)$.

2. **Spatial configuration:** Smaller interzonal distances increase $\mathbb{E}\big(f(d_{ij})\big)$, raising accessibility overall.

The covariance term is further shaped by the spatial distribution of opportunities. If opportunities were uniformly or randomly distributed, this component would vanish, and accessibility would depend only on distance.

Impedance Functions

The impedance function $f(d_{ij})$ captures travel disutility between origin i and destination j, measured in terms of distance, time, cost, or generalised cost (Graham and Gibbons, 2019). Common specifications include negative exponential, power, logarithmic, and Gaussian forms, with parameters typically calibrated on observed travel flows to recover distance-decay sensitivities. Alternative forms have been developed for specific applications, such as healthcare accessibility (Ma et al., 2018) or mode-specific access (e.g., cycling) (Li et al., 2020).

In this research, we adopt the negative exponential form $f(d_{ij}) = \exp(-\beta d_{ij})$, with β as the decay parameter, since it is mathematically consistent with the random utility model framework (Anas, 1983; Miller, 2020). Figure SI3 illustrates the impact of varying β from 0.01 to 0.05: larger β values increase decay, so accessibility decreases more rapidly as β rises. Reported values of β in the literature vary widely depending on context, spatial scale, and travel mode, from as low as 0.005–0.01 for car-based accessibility at the European scale, to 0.05–0.07 for national or regional applications, and up to 0.2–0.3 in local, service-specific studies (e.g., healthcare or convenience shopping) (Stepniak et al., 2013; Schürmann et al., 2002; Reggiani et al., 2011; Geurs and Ritsema van Eck, 2001; Handy and Niemeier, 1997; Haynes et al., 2003).

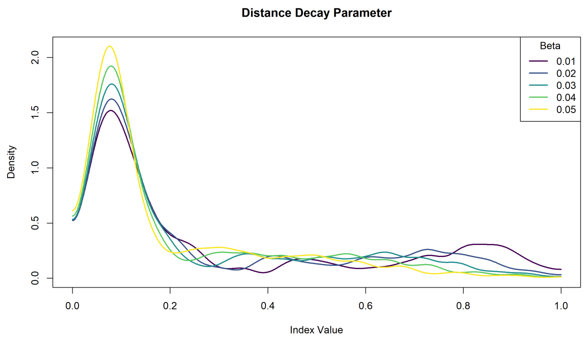

Figure SI3: Effect of the decay parameter: $\beta = 0.01$ to $\beta = 0.05$.

Mass Measures

In gravity models, mass measures capture the attractiveness of destinations. Employment and population are central to this study.

From equation (SI12), the effect of mass decomposes into:

1. the mean mass across all destinations (constant across zones), and

2. the covariance between mass and impedance (varying by zone).

When the spatial distributions of two mass measures (e.g., population and employment) are similar, their covariance structures are also similar, leading to comparable accessibility outcomes. Divergences in distribution, however, generate meaningful differences in accessibility patterns.

Decomposition of the Random Utility Measure

In a random utility maximisation (RUM) framework, accessibility is measured as the expected maximum utility of the set of feasible alternatives. Under the multinomial logit model, this is given by the well-known *logsum* measure:

$$A^{ip} = \ln\left(\sum_{j \in L^{ip}} e^{\beta Z_j}\right), \tag{SI13}$$

where L^{ip} is the set of feasible destination alternatives for individual i to place p, and βZ_j is the systematic utility of alternative j. Here, β is a vector of parameters and Z_j is a vector of explanatory variables such as travel time, cost, or service quality (Concha, 2018).

Equation (SI13) shows that the accessibility measure increases with both the number of alternatives and their quality. The contribution of each option j depends on the sign and magnitude of βZ_j:

- If $\beta Z_j \to -\infty$ (e.g., very high travel costs or poor service), then

$$\lim_{\beta Z_j \to -\infty} e^{\beta Z_j} = 0,$$

and option j has negligible effect on A^{ip}.

- If $\beta Z_j > 0$ (e.g., high service quality or low cost), then $e^{\beta Z_j} > 1$, and option j makes a disproportionately large contribution to accessibility.

The RUM-based logsum measure accounts for both the relative quality of alternatives and the trade-offs between them. Central zones typically exhibit higher accessibility because they offer a larger number of low-cost or high-utility travel options to a larger set of destinations. It is important to note that (SI13) is the closed-form logsum that arises under the multinomial logit model due to the assumption of Gumbel-distributed error terms. In more general random utility models (e.g., nested logit, cross-nested logit, mixed logit, or probit), accessibility can still be interpreted as expected maximum utility, but the corresponding expressions either differ in form (nested logit) or require simulation rather than a closed-form solution (mixed logit, probit).

SI Appendix C Comparing Accessibility

The four clusters identified through k-means clustering applied to the triplet of accessibility scores are shown in Figure SI4. When mapped, they form near-concentric bands across London, indicating a systematic pattern in which the measures move together despite their different constructions. Clustering, therefore, captures broad agreement in spatial structure. This clustering exercise is used to present the cluster-wise analysis of the relationship between accessibility and deprivation in this paper.

Figure SI5 maps the residuals from the spline fits in the pairwise comparisons between accessibility measures. Panel SI5(a) (cumulative vs. gravity) shows residuals scattered around zero with no coherent spatial pattern, thereby consistent with their near-linear agreement. Panels SI5(b) and SI5(c) (pairs with RUM) show a systematic geography: in central and extreme peripheral MSOAs, the cumulative and gravity measures assign higher accessibility than RUM (positive residuals when the Y-axis measure is cumulative or gravity); in a band surrounding the centre, RUM assigns higher accessibility (negative residuals for the Y-axis measure). This implies that RUM compresses accessibility at the highest-access and lowest-access extremes while elevating intermediate areas, which helps explain why it differentiates neighbourhoods less sharply in dense cores.

SI Appendix D Instrument Assumptions and Robustness Tests

Junction Severity Ratio: Construction Details

The JSR is defined as the ratio of serious accidents to total reported accidents at junctions during peak hours, with two important design choices made to improve the plausibility of exogeneity of the instrument (a) floating catchment area (r=2km) aggregation to smooth idiosyncratic spatial anomalies (b) time lagging and pooling the accident data 2004 - 2015 data to create time separation between the instrument and the outcome variable. Further, as a ratio of serious to total accidents, the JSR does not mechanically scale with traffic volumes, improving the plausibility of exogeneity, as traffic flows

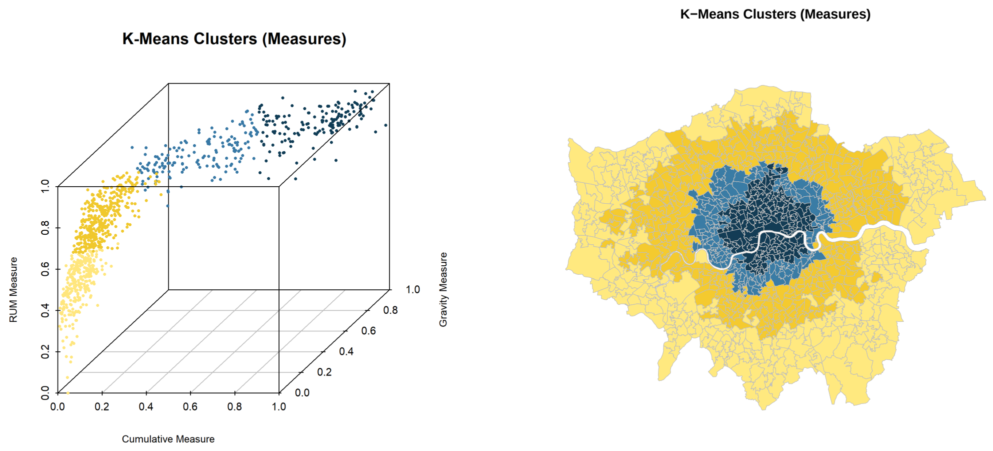

SI4(a) k-means in accessibility measure space

SI4(b) Clusters mapped to 2011 MSOAs

Cluster A Cluster B Cluster C Cluster D

Figure SI4: Clustering of accessibility measures across London. Four clusters were identified as the optimal k using both elbow and silhouette methods, showing systematic co-movement across measures and a near-concentric spatial structure.

are related to local economic activity and deprivation. Since the JSR is representative of road-network complexity, it does not have a direct spatial link with deprivation. Severe traffic accidents are rare events; therefore are unlikely to affect land values or gentrification patterns systematically. Finally, since the JSR includes accidents that occur only during peak periods, it limits the influence of crashes associated with substance abuse or crime, which may be linked with deprivation. Remaining threats to instrument validity are examined explicitly in this section.

Threats to Exclusion

We start by discussing three potential concerns related to the construction of the Junction Severity Ratio (JSR) instrument. One arises from quality concerns arising from the STATS19 data, the second is the influence of driver behaviour and risk avoidance on the JSR, and the third is that safety-related infrastructure may potentially create a direct link between the JSR and deprivation. We outline why these issues are unlikely to undermine the validity of the instrument, while acknowledging remaining limitations.

(a) Under-reporting of accidents in STATS19 Data

The JSR is constructed using STATS19 police-reported collision data. In London, STATS19 data are collected by two police forces: the Metropolitan Police Service, which covers 32 of the 33 local authority districts (LADs) in the study area, and the City of London Police, which covers the City of London. While STATS19 represents the official source of road collision data in Great Britain, it is documented in the Background Quality Report that collisions, particularly those involving only *slight* injuries (casualty classes in the dataset are coded using three values, 1 = fatal, 2 = serious, 3 = slight), are under-reported to the police.

Severe accidents are unlikely to be systematically under-reported, as collisions involving fatalities or serious personal injuries typically require police attendance and formal reporting. By contrast, *slight* accidents may be under-reported, particularly where there is no legal obligation to notify the police. This under-reporting would mechanically reduce the denominator of the ratio and could therefore inflate the measured value of the JSR, representing a potential source of measurement error.

We further note that, although STATS19 data collection follows a standardised process, reporting practices may vary subtly across police forces or locations, particularly for *slight* injuries. While our use of a floating catchment area aggregation, constructing the JSR within a 2 km radius around each MSOA centroid, may smooth some idiosyncratic reporting variation at individual junctions, we acknowledge

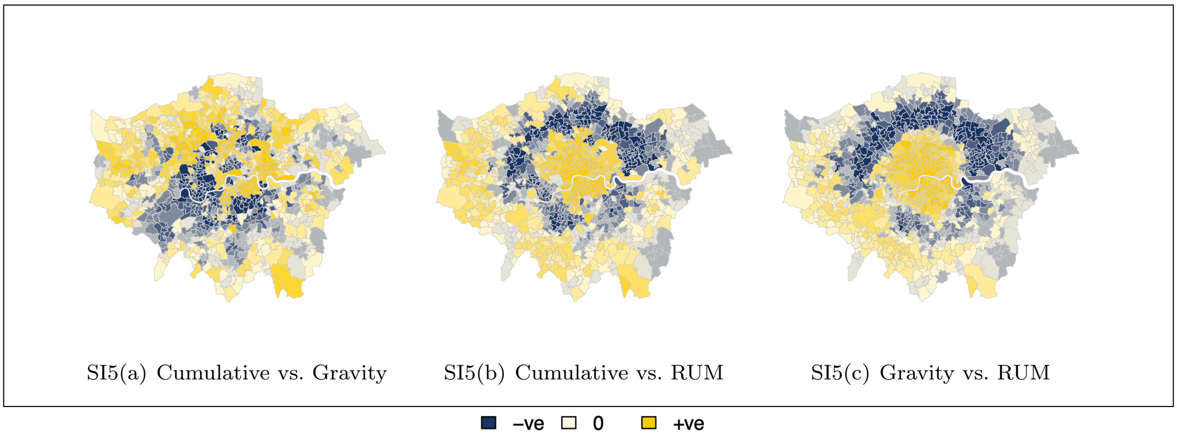

SI5(a) Cumulative vs. Gravity SI5(b) Cumulative vs. RUM SI5(c) Gravity vs. RUM

Figure SI5: Residuals from spline regressions between accessibility measures, mapped at the MSOA level. Positive (yellow) areas indicate the Y-axis measure is higher than predicted given the X-axis measure; negative (dark blue) areas indicate the opposite; white indicates close agreement. The maps reveal minimal systematic divergence for cumulative vs. gravity (SI5(a)), but coherent bands of disagreement for the pairs involving RUM (SI5(b), SI5(c)), concentrated in central MSOAs, a surrounding intermediate band, and the extreme periphery.

that residual under-reporting of slight accidents remains a limitation of the data.

(b) Driver behaviour and risk avoidance

A second concern is that the JSR may reflect driver behaviour, such as risk avoidance of particular intersections with poor safety records, rather than the exogenous design features of the road network. This would be problematic if such behaviour were systematically correlated with deprivation outcomes, reflected in the outcome variable, IMD.

Two features of the instrument design mitigate this concern. First, the JSR is spatially aggregated using a floating catchment area approach rather than being defined at the level of individual junctions. By aggregating accident severity over a 2 km radius, the instrument reduces the influence of highly localised, idiosyncratic collision histories that may be salient to individual drivers. For driver behaviour to bias the instrument, it would require systematic changes in risk-taking or route choice as drivers move through areas of differing deprivation at this broader spatial scale, which we view as less plausible.

Second, the JSR is constructed using pooled accident data from an earlier period (2004–2015), while deprivation outcomes are measured in 2019. This temporal separation further weakens any direct link between contemporaneous driver behaviour and later deprivation outcomes. Past collision severity is therefore unlikely to reflect behavioural responses that are systematically correlated with future socio-economic conditions.

More broadly, the highway safety literature emphasises that observational crash data reflect a selected subset of road users, with recorded collisions disproportionately involving higher-risk drivers and contexts rather than the full population of drivers (Mannering et al., 2020). This suggests that crash-based measures may be influenced by behavioural selection effects that are not fully mitigated by aggregation or temporal lagging, as these processes are intrinsic to collision data. Nevertheless, the objective of this paper is not to estimate causal effects of junction design or safety on individual crash risk, but to make use of persistent, spatially aggregated variation in the JSR. At this aggregate scale, and given the lag and spatial smoothing employed, such selection effects are unlikely to provide a direct pathway through which the JSR would affect deprivation outcomes.

(c) Safety Related Infrastructure Investments

A further potential concern is that there is a link between the JSR and deprivation outcomes through safety-related infrastructure investment. This would be true if safety-related infrastructure were targeted systemically based on deprivation. In the London context, however, such a channel is unlikely. The choice of aggregation, using a 2km floating catchment area (FCA) aggregation, was explicitly intended to smooth over highly localised, potentially deprivation-linked interventions at individual junctions. By aggregating

junction characteristics over a broader spatial catchment, the JSR is insulated from idiosyncratic safety upgrades at specific intersections that could plausibly be correlated with neighbourhood-level socio-economic trends. Further, safety-related investment in London is governed by a centralised institutional structure that prioritises collision risk rather than borough characteristics or neighbourhood deprivation. Transport for London (TfL), established in 2000, is responsible for the Transport for London Road Network (TLRN) and leads city-wide junction safety programmes. Initiatives such as Better Junctions (2012), which prioritised approximately 100 junctions, and Safer Junctions (2017), which prioritised 73 high-risk junctions across London, explicitly target locations with elevated accident rates irrespective of borough boundaries or local socio-economic conditions. Finally, the scale of targeted safety investments is small relative to the overall network and to the broader patterns of junction design complexity captured by the JSR, implying that if these effects exist, they are minimal in this setting.

Robustness Checks on Aggregation Method

We provide some robustness checks on the choice of instrument aggregation radius. The radius of 2 KM is chosen to reflect a neighbourhood-scale environment: London MSOAs vary substantially in physical size, and when treated as equivalent-area circles, their radii range from 0.31 to 2.67 km (central 80%: 0.41–0.95 km). A 2 km radius is therefore larger than a typical MSOA while remaining local in scope, and it avoids the broader spatial aggregation and risks of reduced variability in the instrument implied by larger catchments (e.g. 5–10 km). While radius = 2km was chosen for the baseline results presented in this research, we provide the results for two more radii to show the effect of the aggregation.

Table SI2: IV (2SLS) regression results: accessibility measures and IMD by buffer distance

**Accessibility**	**1km**	**1.5km**	**2km**	**2.5km**	**3km**
Cumulative	-1.208	-1.200*	-1.297**	-1.033**	-0.741**
	(0.810)	(0.505)	(0.393)	(0.337)	(0.281)
Gravity	-0.748	-0.792*	-0.861***	-0.688**	-0.499**
	(0.486)	(0.324)	(0.252)	(0.218)	(0.185)
RUM	-2.910	-2.713*	-3.234**	-2.470**	-1.728*
	(2.261)	(1.331)	(1.195)	(0.931)	(0.706)

Notes: Estimates are IV (2SLS) coefficients using the Junction Severity Ratio as the instrument. Robust standard errors in parentheses. *** $p < 0.001$, ** $p < 0.01$, * $p < 0.05$.

In Table SI2 we present the 2SLS results for the aggregated regressions, that is, including all 983 MSOAs in the study area, against the weighted IMD. The results indicate that at 2 KM, there is sufficient variation in the IV to find statistically significant and consistent results across all three measures of accessibility. As smaller aggregation radii are adopted, estimate precision declines, reflecting a weakening of the first-stage relationship between the instrument and accessibility. Conversely, at larger aggregation radii, identifying variation in the instrument is increasingly smoothed out, reducing statistical power.

In Table SI3 we present the 2SLS results for the aggregated regressions, including all 983 MSOAs in the study area, against specific domains of the IMD - the Income, Employment, Housing, and Health. The effects of aggregation radius choice, as shown in Table SI2, are especially apparent for the Income and Employment domains. As the radius decreases, the first stage weakens, while at larger radii estimate precision declines, as broader aggregation attenuates within-LAD variation. For the Education domain, consistent, statistically significant, negative effects are found across the aggregation radii shown. Finally, for the Health domain, no significant results are found across all aggregation radii.

In Table SI4 we present the 2SLS results for the aggregated regressions, including all 983 MSOAs in the study area, against the remaining IMD domains, including the crime, housing, and living domains. For the Crime domain, no relationships are found across all the aggregation radius choices. For the Housing domain, and the Living domain, consistent, statistically significant negative effects are found across the aggregation radii shown.

Figure SI6 provides a visual comparison of the JSR with the IMD. While visual inspection is not sufficient to determine exclusion, this is presented to supplement the discussion on the exclusion restriction presented, along with the overidentification tests reported in this research. Importantly, the figure shows no systematic relationship between centrality and JSR across London's MSOAs. Figure SI7 illustrates the effect of different aggregation radii on the behaviour of the JSR across London's MSOAs. Larger

Table SI3: IV (2SLS) results: Income, Employment, Education and Health domains by buffer distance

Domain	#	1km	1.5km	2km	2.5km	3km
**Income**	G	-1.302<br>(0.780)	-1.227*<br>(0.487)	-1.409***<br>(0.384)	-1.106***<br>(0.332)	-0.805**<br>(0.282)
	C	-0.807<br>(0.467)	-0.811**<br>(0.312)	-0.936***<br>(0.245)	-0.737***<br>(0.214)	-0.543**<br>(0.186)
	R	-3.138<br>(2.204)	-2.775*<br>(1.284)	-3.514**<br>(1.180)	-2.643**<br>(0.917)	-1.878**<br>(0.704)
**Employment**	G	-1.008<br>(0.743)	-0.949*<br>(0.463)	-1.070**<br>(0.365)	-0.864**<br>(0.321)	-0.565*<br>(0.278)
	C	-0.625<br>(0.449)	-0.627*<br>(0.299)	-0.711**<br>(0.236)	-0.576**<br>(0.210)	-0.381*<br>(0.185)
	R	-2.429<br>(1.982)	-2.145<br>(1.159)	-2.668*<br>(1.047)	-2.065*<br>(0.844)	-1.319*<br>(0.668)
**Education**	G	-2.837***<br>(0.778)	-2.980***<br>(0.503)	-3.090***<br>(0.396)	-2.972***<br>(0.352)	-2.707***<br>(0.307)
	C	-1.758***<br>(0.446)	-1.969***<br>(0.304)	-2.053***<br>(0.235)	-1.979***<br>(0.210)	-1.824***<br>(0.187)
	R	-6.835**<br>(2.577)	-6.739***<br>(1.588)	-7.706***<br>(1.493)	-7.103***<br>(1.199)	-6.315***<br>(0.914)
**Health**	G	-0.306<br>(0.700)	-0.363<br>(0.436)	-0.387<br>(0.346)	-0.234<br>(0.312)	0.086<br>(0.271)
	C	-0.190<br>(0.431)	-0.240<br>(0.286)	-0.257<br>(0.227)	-0.156<br>(0.207)	0.058<br>(0.183)
	R	-0.738<br>(1.720)	-0.822<br>(1.021)	-0.965<br>(0.899)	-0.559<br>(0.760)	0.201<br>(0.631)

Notes: Estimates are IV (2SLS) coefficients using the Junction Severity Ratio as the instrument. Robust standard errors are reported in parentheses below coefficients. Accessibility measure codes: 1 = cumulative, 2 = gravity, 3 = random utility. *** $p < 0.001$, ** $p < 0.01$, * $p < 0.05$.

Table SI4: IV (2SLS) results: IMD domains by buffer distance

Dom.	#	1km	1.5km	2km	2.5km	3km
**Crime**	C	-0.084<br>(0.697)	-0.175<br>(0.438)	-0.062<br>(0.338)	0.092<br>(0.291)	0.244<br>(0.257)
	G	-0.052<br>(0.431)	-0.116<br>(0.289)	-0.041<br>(0.224)	0.062<br>(0.194)	0.165<br>(0.174)
	R	-0.204<br>(1.696)	-0.395<br>(1.013)	-0.154<br>(0.850)	0.221<br>(0.687)	0.570<br>(0.588)
**Housing**	C	-3.416***<br>(0.939)	-3.248***<br>(0.613)	-3.256***<br>(0.474)	-2.986***<br>(0.402)	-2.802***<br>(0.343)
	G	-2.116***<br>(0.527)	-2.146***<br>(0.372)	-2.163***<br>(0.285)	-1.989***<br>(0.245)	-1.888***<br>(0.212)
	R	-8.230*<br>(3.552)	-7.344***<br>(2.038)	-8.121***<br>(1.876)	-7.136***<br>(1.461)	-6.535***<br>(1.146)
**Living.**	C	2.898***<br>(0.550)	2.884***<br>(0.354)	2.789***<br>(0.285)	2.883***<br>(0.261)	2.899***<br>(0.233)
	G	1.796***<br>(0.333)	1.906***<br>(0.225)	1.853***<br>(0.183)	1.920***<br>(0.167)	1.953***<br>(0.148)
	R	6.983***<br>(1.615)	6.523***<br>(0.906)	6.955***<br>(0.885)	6.890***<br>(0.716)	6.761***<br>(0.613)

Notes: Estimates are IV (2SLS) coefficients using the Junction Severity Ratio as the instrument. Robust standard errors are reported in parentheses below coefficients. Accessibility measure codes: 1 = cumulative, 2 = gravity, 3 = random utility. *** $p < 0.001$, ** $p < 0.01$, * $p < 0.05$.

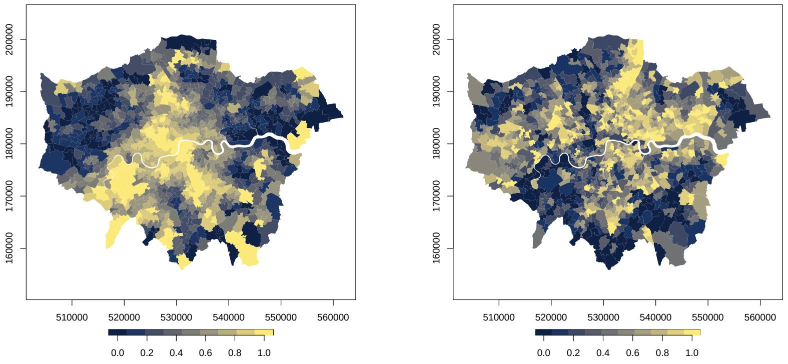

SI6(a) JSR (r=2) at MSOA Level SI6(b) IMD 2019 at the MSOA Level

Figure SI6: Panel (a) displays the floating catchment area JSR with r=2km, while Panel (b) shows the IMD at the MSOA level. Visual inspection does not reveal any clear or systematic spatial correspondence between areas of high or low deprivation alongside the behavior of the instrument. This visual is presented alongside the standard over-identification tests presented in this paper, as visual inspection alone is insufficient to prove the exogeneity of the instrument.

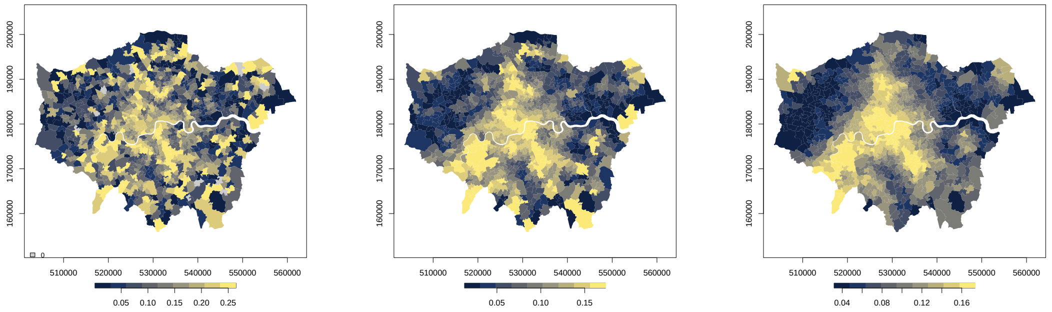

SI7(a) JSR FCA (r = 1) SI7(b) JSR FCA (r = 2) SI7(c) JSR FCA (r = 3)

Figure SI7: Panel (a) displays an FCA with a 1km radius, while Panel (b) displays an FCA with a 2km radius. Panel (b) displays an FCA with a 3km radius. These figures show that at (r=1) there are some 0 values. As the radius grows, the spatial variation of the instrument reduces.

aggregation radii smooth local differences, resulting in reduced variation in the JSR.

SI Appendix E State of Practice in London

Transport for London (TfL) recommends three main methods for conducting a “connectivity assessment,” which plays a central role in strategic planning. TfL uses the term “connectivity” to describe the ease of travel between zones; in this research, we adopt the equivalent term “accessibility.” The three approaches endorsed by TfL are: (A) the Public Transport Access Level (PTAL), which evaluates the availability and quality of the transport network (i.e., transport supply), and two opportunity-based measures, (B) catchment area analysis and (C) travel time mapping, which assess the ability of people to reach destinations through the transport system (Inayathusein and Cooper, 2018; for London, 2015).

Public Transport Access Level (PTAL) — A Measure of Transport Supply

Public Transport Access Level (PTAL) is TfL’s most widely used measure and reflects the supply of public transport services in each zone. PTAL values range from 0 (very poor supply) to 6 (excellent supply). They are derived from four datasets: (1) the locations of trip origins (such as houses, offices, shops), (2) the locations of all stations and stops in London (“Service Access Points,” SAPs), (3) the walk network used to reach these SAPs, and (4) the frequencies of services at the SAPs. Crucially, PTAL does not

account for the destinations that can be reached via the network; instead, it reflects only the density and frequency of nearby services (TfL, 2015). Its simplicity and reliance on readily available data make PTAL attractive for planning, and the method has been exported to other contexts, including the Netherlands, the USA, Australia, New Zealand, and India (Adhvaryu and Kumar, 2021). However, PTAL remains a supply-side measure, providing no direct insight into the opportunities actually accessible from each location.

Catchment Area Analysis

Catchment area analysis provides an opportunity-based perspective by mapping which destinations can be reached within pre-defined travel time thresholds from each zone centroid. Variations of this method are common: (a) mapping the catchment areas themselves with labelled travel times, (b) reporting socio-economic information within the catchment (such as the number of jobs by type or the income profile of residents), (c) listing services (such as hospitals, colleges, or town centres) within reach, and (d) comparing catchments across scenarios (e.g., by mode, time of day, or policy intervention). Catchment analysis can focus on a single zone or be applied across all zones in the study area. Conceptually, this method corresponds to cumulative-count accessibility measures, as discussed in this research.

Travel Time Mapping

The third method, travel time mapping, is used by TfL for strategic planning. This approach calculates travel times from one origin zone to every other zone, for a specific mode, destination type, and/or time period. The results are typically visualised as maps that display isochrones or colour gradients to represent travel times across the city. While similar in purpose to catchment analysis, travel time mapping provides a more flexible representation of the relative accessibility of zones and is especially useful for communicating differences in accessibility across scenarios.

Taken together, these three methods illustrate TfL's dual approach: PTAL as a supply-side metric that focuses on service provision, and catchment analysis and travel time mapping as opportunity-based measures that consider the destinations residents can actually reach. This mix highlights the distinction between supply-side and opportunity-based perspectives in accessibility research and situates our study within broader London planning practice. Figure SI8 illustrates the Lorenz curves using the cumulative measure, the gravity measure and RUM measure, which are constructed as described in this paper, and the PTAL scores as defined by TfL. This figure illustrates how the choice of accessibility measures affects conclusions on transport equity.

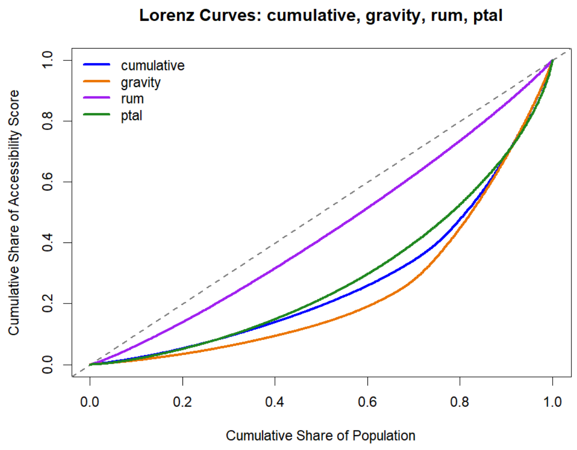

Figure SI8: Lorenz curves comparing the distribution of public transport accessibility across London under four measures: cumulative (Gini = 0.727), gravity (Gini = 0.759), random utility (RUM, Gini = 0.563), and PTAL (Gini = 0.710). The RUM measure shows the most equitable accessibility distribution, while the gravity measure exhibits the highest inequality. These differences illustrate how the choice of accessibility measure fundamentally shapes observed equity outcomes.

SI Appendix F IMD by Domain

This section provides a detailed description of the component domains that form the English Index of Multiple Deprivation (IMD) 2019. Each domain aggregates non-overlapping indicators that capture specific dimensions of deprivation at the Lower-layer Super Output Area (LSOA) level. The corresponding spatial distributions of these domains across London are shown in Figure SI9.

Income Domain

Measures deprivation related to low income, capturing adults and children in families receiving means-tested benefits or low-income tax credits, as well as asylum seekers supported by the Home Office.

Employment Domain

Captures involuntary exclusion from the labour market. Indicators include claimants of unemployment, incapacity, and disability-related benefits, as well as those on Universal Credit with work-search requirements.

Education, Skills and Training Domain

Reflects educational disadvantage in both children and adults.
Children and Young People Sub-domain: Attainment at Key Stages 2 and 4, school absences, post-16 participation, and progression to higher education.
Adult Skills Sub-domain: Proportion of adults with low or no qualifications and limited English language proficiency.

Health Deprivation and Disability Domain

Measures premature mortality, illness, and disability. Indicators include years of potential life lost, comparative illness and disability ratios, emergency hospital admissions, and prevalence of mood and anxiety disorders.

Crime Domain

Captures risk of personal and material victimisation based on recorded rates of violence, burglary, theft, and criminal damage.

Barriers to Housing and Services Domain

Assesses physical and financial access to housing and local services.
Geographical Barriers: Road distance to key amenities (GP, schools, shops, post office).
Wider Barriers: Overcrowding, homelessness, and housing affordability.

Living Environment Domain

Reflects housing quality and external environmental conditions.
Indoors Sub-domain: Proportion of homes lacking central heating or failing the Decent Homes standard.
Outdoors Sub-domain: Air quality and road traffic accidents involving pedestrians and cyclists.

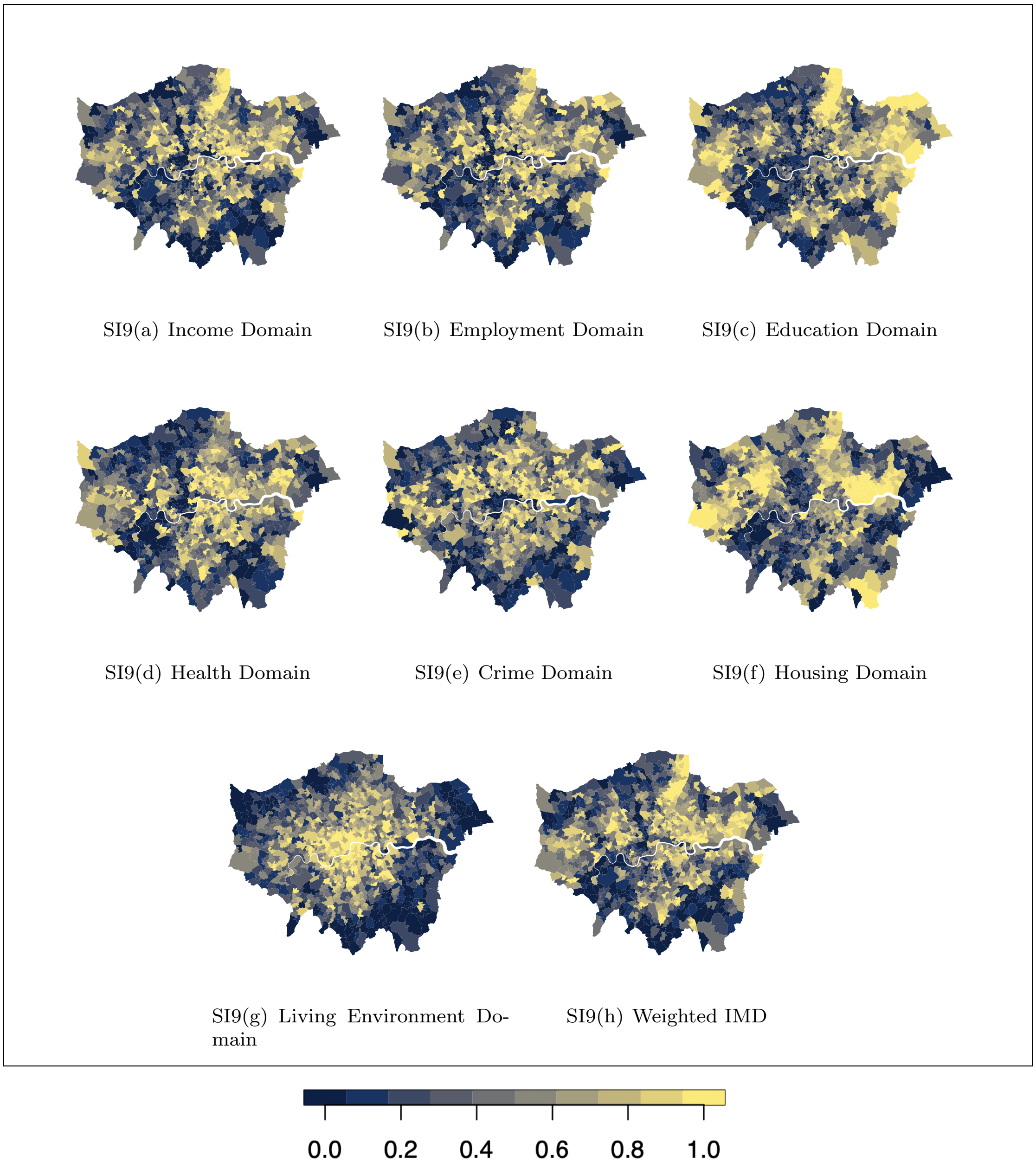

SI9(a) Income Domain

SI9(b) Employment Domain

SI9(c) Education Domain

SI9(d) Health Domain

SI9(e) Crime Domain

SI9(f) Housing Domain

SI9(g) Living Environment Domain

SI9(h) Weighted IMD

Figure SI9: 2019 IMD domains for London MSOAs. The figure visualises each of the seven component domains, as well as the overall weighted IMD score, corresponding to the descriptions provided in this appendix.

References

Adhvaryu, B. and Kumar, S. (2021). Public transport accessibility mapping and its policy applications: a case study of lucknow, india. *Case studies on transport policy*, 9(4):1503–1517.

Anas, A. (1983). Discrete choice theory, information theory and the multinomial logit and gravity models. *Transportation Research Part B: Methodological*, 17(1):13–23.

Ben-Akiva, M. E. and Lerman, S. R. (1985). *Discrete choice analysis: theory and application to travel demand*, volume 9. MIT press.

Bergstrand, J. H. (1985). The gravity equation in international trade: Some microeconomic foundations and empirical evidence. *The Review of Economics and Statistics*, 67(3):474–481.

Concha, F. M. (2018). Microeconomic modeling in urban science. In *Microeconomic Modeling in Urban Science*, chapter 2, pages 21–40. Academic Press.

Dong, X., Ben-Akiva, M., Bowman, J. L., and Walker, J. L. (2006). Moving from trip-based to activity-based measures of accessibility. *Transportation Research Part A: Policy and Practice*, 40(2):163–180.

for London, T. (2015). Assessing transport connectivity in london.

Geurs, K. T. and Ritsema van Eck, J. R. (2001). Accessibility measures: review and applications. evaluation of accessibility impacts of land-use transportation scenarios, and related social and economic impact.

Geurs, K. T. and Ritsema van Eck, J. R. (2006). The impact of accessibility measures on integrated transport–land-use modelling. *Environment and Planning A*, 38(9):1533–1549.

Giannotti, M., Barros, J., Tomasiello, D. B., Smith, D., Pizzol, B., Santos, B. M., Zhong, C., Shen, Y., Marques, E., and Batty, M. (2021). Inequalities in transit accessibility: Contributions from a comparative study between global south and north metropolitan regions. *Cities*, 109:103016.

Graham, D. J. and Gibbons, S. (2019). Quantifying wider economic impacts of agglomeration for transport appraisal: Existing evidence and future directions. *Economics of Transportation*, 19:100121.

Handy, S. L. and Niemeier, D. A. (1997). Measuring accessibility: an exploration of issues and alternatives. *Environment and planning A*, 29(7):1175–1194.

Hansen, W. G. (1959). How accessibility shapes land use. *Journal of the American Institute of planners*, 25(2):73–76.

Hasnine, M. S., Graovac, B., Camargo, L. F. O., and Habib, K. M. N. (2019). A random utility maximization (rum) based measure of accessibility to transit: Accurate capturing of the first-mile issue in urban transit. *Journal of Transport Geography*, 74:313–320.

Haynes, R., Lovett, A., and Sünnenberg, G. (2003). Potential accessibility, travel time, and consumer choice: geographical variations in general medical practice registrations in eastern england. *Environment and Planning A*, 35(10):1733–1750.

Head, K. and Mayer, T. (2014). Gravity equations: Workhorse, toolkit, and cookbook. In *Handbook of international economics*, volume 4, pages 131–195. Elsevier.

Inayathusein, A. and Cooper, S. (2018). London's accessibility indicators: Strengths, weaknesses, challenges. Technical report.

Kapatsila, B., Palacios, M. S., Grisé, E., and El-Geneidy, A. (2023). Resolving the accessibility dilemma: Comparing cumulative and gravity-based measures of accessibility in eight canadian cities. *Journal of Transport Geography*, 107:103530.

Klar, B., Lee, J., Long, J. A., and Diab, E. (2023). The impacts of accessibility measure choice on public transit project evaluation: A comparative study of cumulative, gravity-based, and hybrid approaches. *Journal of Transport Geography*, 106:103508.

Li, A., Huang, Y., and Axhausen, K. W. (2020). An approach to imputing destination activities for inclusion in measures of bicycle accessibility. *Journal of transport geography*, 82:102566.

Ma, L., Luo, N., Wan, T., Hu, C., and Peng, M. (2018). An improved healthcare accessibility measure considering the temporal dimension and population demand of different ages. *International journal of environmental research and public health*, 15(11):2421.

Mannering, F., Bhat, C. R., Shankar, V., and Abdel-Aty, M. (2020). Big data, traditional data and the tradeoffs between prediction and causality in highway-safety analysis. *Analytic methods in accident research*, 25:100113.

Miller, E. J. (2019). Agent-based activity/travel microsimulation: What's next? In Thill, J.-C. and Pereira, R., editors, *The Practice of Spatial Analysis: Essays in Memory of Professor Pavlos Kanaroglou*, pages 119–150. Springer, Cham.

Miller, E. J. (2020). Measuring accessibility: Methods and issues.

Palacios, J. P. (2022). Cumulative versus gravity-based accessibility measures: Which one to use? *Findings*, —.

Reggiani, A., Bucci, P., and Russo, G. (2011). Accessibility and impedance forms: empirical applications to the german commuting network. *International Regional Science Review*, 34(2):230–252.

Schürmann, C., Spiekermann, K., and Wegener, M. (2002). European accessibility and peripherality: Concepts, models and indicators.

Shannon, C. E. (1948). A mathematical theory of communication. *The Bell system technical journal*, 27(3):379–423.

Shen, Q. (1998). Location characteristics of inner-city neighborhoods and employment accessibility of low-wage workers. *Environment and planning B: Planning and Design*, 25(3):345–365.

Stepniak, P., Rosik, T., and Komornicki, T. (2013). Accessibility patterns: Poland case study. *Europa Xxi*, 24:77–93.

Wilson, A. G. (1971). A family of spatial interaction models, and associated developments. *Environment and Planning A*, 3(1):1–32.
